# Biocontrol Potential of Raw Olive Mill Waste Against *Verticillium dahliae* in Vegetable Crops

**DOI:** 10.3390/plants14060867

**Published:** 2025-03-10

**Authors:** Stefanos K. Soultatos, Anastasia Chatzaki, Panagiotis A. Karas, Anastasia A. Papadaki, Georgios S. Kalantzakis, Georgios Psarras, Dimitrios E. Goumas, Dimitrios G. Karpouzas, Emmanouil A. Markakis

**Affiliations:** 1 Department of Agriculture, School of Agricultural Sciences, Hellenic Mediterranean University, Stavromenos, 71004 Heraklion, Greece; stefanoss1313@gmail.com (S.K.S.); dgoumas@hmu.gr (D.E.G.); 2 Institute of Olive Tree, Subtropical Crops and Viticulture, Hellenic Agricultural Organization—DIMITRA, Agrokipio, 73100 Chania, Greeceapapadaki@hmu.gr (A.A.P.); kalantzakis@elgo.gr (G.S.K.); psarras@elgo.gr (G.P.); 3 Department of Biochemistry and Biotechnology, University of Thessaly, Viopolis, 41500 Larisa, Greece; karaspan@yahoo.com (P.A.K.); dkarpouzas@uth.gr (D.G.K.)

**Keywords:** bioassays, disease suppression, microbiome, protection, verticillium wilt

## Abstract

Verticillium wilt caused by the soil-borne fungus *Verticillium dahliae* causes severe losses to a broad range of economically important crops worldwide. Chemical disease management is ineffective; thus, alternative control strategies are needed. Olive-producing countries face the challenge of managing olive mill wastewater (OMW) in an environmentally friendly and agronomically beneficial manner. The proper use of OMW supported by scientific research has been proposed as a valuable means for successful disease management. In this respect, we tested whether soil application of raw OMW can protect vegetable crops against *V. dahliae* and investigated the potential disease-suppressive mechanisms. OMW inhibited significantly fungal growth, sporulation, hyphae width, and conidial and microsclerotial germination *in vitro*, and these effects were dose-dependent. Moreover, the addition of OMW in the soil provided sufficient protection of eggplant and tomato against *V. dahliae* in planta. The high OMW-conferred protection of eggplant was gradually decreased, possibly due to the decreased phenolic content in OMW over time. Bioassays with sterilized soil substrate and OMW, along with isolated microbial strains, revealed that soil- and OMW-originated microbes had no role in disease suppression. Moreover, split-root set-ups suggested a non-systemic OMW-induced resistance mechanism. Root-drench application of OMW in eggplant and tomato plants did not cause significant alterations in the structure of the plant microbiome that could be associated with disease suppressiveness.

## 1. Introduction

Verticillium wilt caused by the soil-borne fungus *Verticillium dahliae* Kleb. is among the most devastating plant diseases affecting more than 350 plant species worldwide [1]. The disease causes severe losses to several economically important crops including vegetables, fruits, ornamentals, oilseed crops, fibre crops and woody perennials, and is also capable of invading several weeds [2]. Due to its broad host range and capacity to survive for up to fifteen years in the soil, forming resistant structures named ‘microsclerotia’ and benefiting from the lack of effective fungicides, *V. dahliae* is especially difficult to manage [3]. Therefore, management strategies are focused on preventive measures such as solarization of heavily infested soils, the use of resistant/tolerant genotypes, attempts for biological control practices including the use of compost soil amendments or direct application of microorganisms with proven protective activity against the pathogen, or combinations of the above.

On the other hand, agricultural-oriented countries produce massive quantities of agro-industrial by-products and face the challenge of managing such materials in an environmentally friendly and agronomically efficient manner. This is the case for the significant amounts of olive mill wastewater (OMW) that are generated annually during the olive oil extraction process in major olive-producing countries [4]. OMW constitutes a serious environmental problem in the Mediterranean regions that account for approximately 95% of the world's olive oil production due to its high organic load, phytotoxicity and antimicrobial effects. Amongst other agro-industrial by-products, OMW has been proposed to be used in beneficial ways as biofertilizers in several crops or biological protectants against a broad range of soil-borne and aerial pathogens [4].

In the last two decades, OMW has been verified as a valuable source of resistance for plants against numerous pathogens. With respect to verticillium wilt, several studies have demonstrated the suppressive effect of OMW-derived composts, extracts or microorganisms against *V. dahliae in vitro* and in vegetable crops in planta [5,6,7,8,9]. Phenolic substances are among the abiotic factors that have been found to contribute to the suppressive activity of OMW-associated compost amendments and extracts against *V. dahliae* [5,7,9], whereas the biological nature of the disease-suppressive mechanism has also been addressed by isolating and screening certain microbial strains [8]. However, the potential suppressive effect of raw OMW material against verticillium wilt in planta has not been investigated previously.

Land spreading of OMW is an alternative management practice in several Mediterranean countries that have introduced specific legislation, having several beneficial effects on crops when applied in proper dosage and timing [4,10]. OMW application in soil induces changes in soil physical and chemical properties with cascading effects on the structure of the soil bacterial communities [10,11]. Moreover, OMW is a microbial-rich material containing many distinct groups of microorganisms including numerous bacterial, yeast and fungal genera [12,13,14]. Despite the microbial nature of OMW and the observed alterations in the structure of soil bacterial communities upon OMW application, little is known regarding the effects of OMW land application on the plant microbiome of treated plants and particularly on its role in disease suppression. Therefore, the main objectives of the present study were to (i) evaluate the suppressive effect of raw OMW against verticillium wilt in vegetable crops, (ii) investigate the disease-suppressive mechanisms and (iii) study the potential effect of OMW on the eukaryotic and prokaryotic plant microbiome in OMW-treated plants with respect to disease suppression.

## 2. Results

### 2.1. Chemical Properties of OMW

The effects of heat sterilization of OMW at the beginning of the experiments (July 2019) on moisture, chemical composition in terms of plant macro- and micronutrients and total phenolic content are shown in Supplementary Table S1. Only slight differences were observed for moisture and most of the macro- and micronutrients, whereas total phenolic content was significantly decreased in the heat-sterilized OMW sample in July 2019. Furthermore, total phenol content in non-sterilized OMW in October 2020 was significantly lower than that measured in non-sterilized and sterilized OMW in July 2019, indicating a pattern of decreasing phenolic load in OMW over time.

### 2.2. Effects of Various Concentrations of OMW on V. dahliae In Vitro

**Figure 1:**
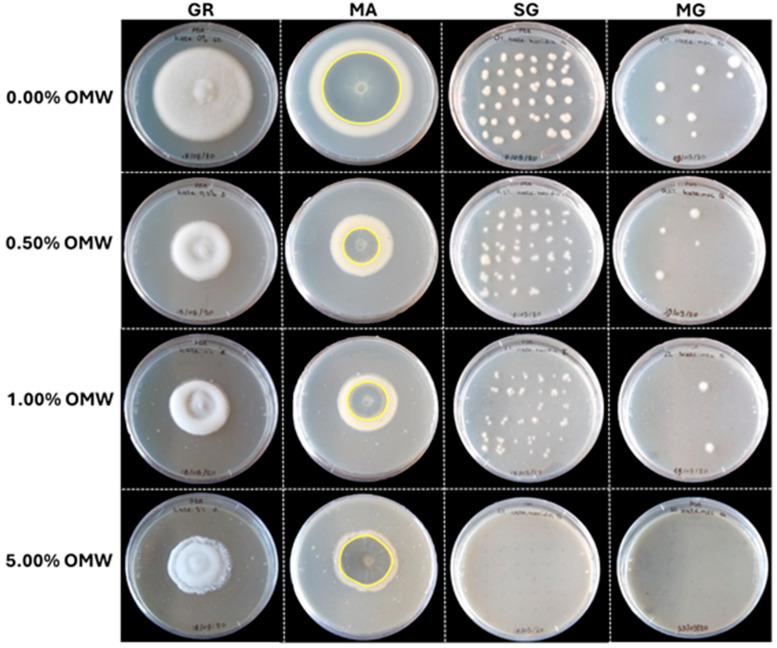
Effect of sterilized olive mill wastewater (OMW) added at various concentrations (0.00%, 0.50%, 1.00% and 5.00%) in PDA, on fungal parameters (**GR:** growth rate; **MA:** microsclerotial area; **SG:** spore germination; **MG:** microsclerotium germination) of *Verticillium dahliae*. Yellow lines on the underside of PDA cultures indicate the border of the microsclerotial area among the different treatments *in vitro*.

Apart from the microsclerotial area, all the other fungal parameters of *V. dahliae* were affected significantly by OMW, mostly when OMW was incorporated at higher concentrations in PDA cultures (i.e., 0.50%, 1.00% and 5.00%) (Table 1, Figure 1). Interestingly, *V. dahliae’s* growth rate, sporulation, hyphae width and microsclerotia germination in PDA supplemented with 5.00% OMW were decreased by 36.20%, 77.90%, 38.21% and 84.09%, respectively, compared to control (0.00% OMW), whereas conidial germination was eliminated. In addition, OMW concentration was negatively correlated with growth rate (df = 46, r = −0.465, *p* ≤ 0.001), sporulation (df = 46, r = −0.388, *p* ≤ 0.01), spore germination (df = 46, r = −0.899, *p* ≤ 0.001) and microsclerotium germination (df = 46, r = −0.533, *p* ≤ 0.001), whereas no significant correlation was identified between OMW content and hyphae width (df = 46, r = −0.185, *p* = 0.210) or microsclerotial area (df = 46, r = 0.007, *p* = 0.962).

### 2.3. Suppression of Verticillium Wilt of Eggplant in Experiment I

Verticillium wilt symptoms on eggplant in experiment Ι (set up in November 2019) started 12 days post-inoculation (dpi) in plants treated with high *V. dahliae* inoculum density (V.d. high). Disease severity progressed rapidly over time in V.d. high-treated plants, finally reaching 68.49% at 30 dpi (Supplementary Figure S1). Plants treated with high inoculum density, previously treated with TRIANUM-P (V.d. high + TRIANUM-P), as well as those inoculated with low inoculum density (V.d. low) showed less prominent symptoms and slower disease development over time, reaching final disease severity values of 33.45% and 34.25%, respectively (Table 2, Supplementary Figure S1). Plants treated with OMW, inoculated with either high (V.d. high + OMW) or low (V.d. low + OMW) inoculum density, showed very slow disease progression, and values of disease severity never exceeded 4.06% or 1.90%, respectively, over the whole assessment period.

All the disease parameters in OMW-treated plants inoculated with high *V. dahliae* inoculum density were significantly lower compared to the respective non-OMW-treated ones. This was also the case for most of the parameters in OMW-treated plants inoculated with low inoculum density (Table 2). In addition, disease incidence, final disease severity and relative AUDPC values in V.d. high + OMW-treated plants were significantly lower than in V.d. high + TRIANUM-P-treated plants. Moreover, V.d. high + OMW-treated plants developed significantly higher fresh weight compared to the V.d. high-treated ones. All the above indicate a strong protective activity of OMW against the pathogen in planta, which is superior to even the commercial fungicide TRIANUM-P.

Interestingly, increased fungal growth was observed on the soil surface in the OMW-treated pots with white-green *Trichoderma*-like colonies being evident visually after one week post-OMW application, whereas such colonies were not developed in controls (water-treated pots). Five fungal strains isolated from the OMW-treated soil substrate (designated as KX1, KX2, KX3, KX6 and KX7) and one isolated from vessels of an asymptomatic *V. dahliae*-inoculated eggplant that had received OMW treatment (designated as KF8) in experiment I were evaluated for their protective activity against the pathogen in planta in the following experiment.

### 2.4. Suppression of Verticillium Wilt of Eggplant in Experiment II

Likewise, verticillium wilt symptoms on eggplant in experiment II (set up in April 2020) progressed rapidly in most treatments but this was not the case for OMW-treated plants (Supplementary Figure S2). In particular, disease severity in OMW-treated plants (treatments V.d. high + OMW, V.d. high_Steril. Soil + OMW and V.d. low + OMW) was significantly lower compared to their respective controls (V.d. high and V.d. low) at 18, 25 and 32 dpi. Contrarywise, disease severity in plants treated with individual microbial strains or a mix of them (V.d. high + KX1, V.d. high + KX2, V.d. high + KX3, V.d. high + KX6, V.d. high + KX7, V.d. high + KF8 or V.d. high + MIXED) did not differ significantly compared to controls. V.d. high + TRIANUM-P-treated plants showed intermediate disease progress over time with significantly lower disease severity than controls at 18 dpi; however, disease severity increased steadily at 25 and 32 dpi, reaching non-significant values compared to control (Supplementary Figure S2).

Overall, all disease parameters in OMW-treated plants were significantly lower, and these plants developed significantly higher height and fresh weight than controls, indicating a strong protective activity of OMW against *V. dahliae* (Table 3). Interestingly, re-isolations performed from OMW-treated plants inoculated with *V. dahliae* revealed several fungi (mostly *Trichoderma*-like strains) emerging either solely from a xylem chip or simultaneously with *V. dahliae* from the same chip. Such fungal contaminants were not observed in re-isolations performed from positive control plants (V.d. high- and V.d. low-treated), indicating a differential texture of endophytic fungal inhabitants in OMW-treated plants.

TRIANUM-P-treated plants differed significantly over controls in terms of relative AUDPC and qPCR only, whereas they did not differ significantly in plant growth parameters, suggesting a lesser efficient protective activity of the commercial biofungicide TRIANUM-P against the pathogen. The equal protective activity of OMW applied in either non-sterilized (V.d. high + OMW) or sterilized soil substrate (V.d. high_Steril. Soil + OMW), along with the ineffective protection of plants by individual soil-originated microbial strains and their mixture, indicates an unlikely association of soil microbiome with disease-suppressive mechanisms in planta.

### 2.5. Suppression of Verticillium Wilt of Tomato in Experiment III

Verticillium wilt symptoms on tomato in experiment IIΙ (set up in June 2020) started at 11 dpi in both OMW-treated plants and controls inoculated with *V. dahliae*; however, the latter plant group developed a comparatively rapid disease progress over time. Disease severity in OMW-treated plants was significantly lower compared to the non-treated controls at all observation time points (Supplementary Figure S3). Moreover, all the evaluated disease parameters (apart from mortality) in OMW-treated plants were significantly lower than those in the non-treated ones, while the fresh weight of OMW-treated plants was significantly increased (Table 4). Interestingly, the significantly lower values of *V. dahliae* isolation ratio and qPCR quantification in OMW-treated plants compared to controls suggest the decreased vascular colonization by the pathogen due to OMW application.

### 2.6. Suppression of Verticillium Wilt of Eggplant in Experiment IV

Verticillium wilt symptoms on eggplant in experiment IV (set up in July 2020) progressed rapidly in positive control plants (V.d.), whereas the differentially treated plants (V.d. + OMW, V.d. + Steril. OMW, V.d./- and V.d./OMW) exhibited slower disease progress. Disease severity in plants treated with sterilized and non-sterilized OMW (V.d. + Steril. OMW and V.d. + OMW, respectively) was significantly lower than in the controls (V.d.) in the single-pot set-ups, with such difference being noticed not earlier than 18 dpi (Supplementary Figure S4). However, none of the estimated disease parameters indicated significant differences between plants treated with sterilized and non-sterilized OMW (Table 5, Supplementary Figure S4), suggesting that disease suppression is not related to the OMW-originated microbiome. Furthermore, the non-significant disease parameters between V.d./- and V.d./OMW-treated plants in the split-root set-ups indicate that disease-suppressive mechanisms are not related to systemic OMW-induced resistance events.

### 2.7. Suppression of Verticillium Wilt of Eggplant in Experiment V

**Figure 2:**
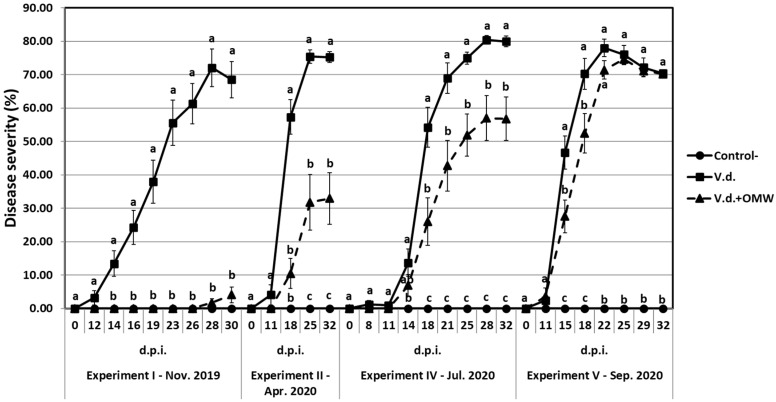
Verticillium wilt disease severity on eggplant mock-inoculated (control-) or inoculated with 20 mL of high (5 × 10^6^ conidia mL^−1^) inoculum density of *Verticillium dahliae*, treated with olive mill wastewater (OMW) or non-treated, in different experiments (I, II, IV and V) conducted at different periods under the same controlled conditions. Each marker represents the mean of 21 plants. Within each experiment, markers followed by the same letter at each observation time point are not significantly different according to Tukey’s HSD test at *p* ≤ 0.05. Vertical bars indicate standard errors.

In contrast to the previous experiments, verticillium wilt symptoms on eggplant in experiment V (set up in September 2020) progressed rapidly in both OMW-treated and non-treated plants inoculated with *V. dahliae*. None of the evaluated disease (apart from relative AUDPC) and plant growth parameters showed statistically significant values between V.d.- and V.d. + OMW-treated plants (Table 6), indicating a lower suppressive efficiency of OMW against *V. dahliae* in experiment V, compared to the previous experiments. A significant difference between disease severity in V.d.- and V.d. + OMW-treated plants was observed at 15 and 18 dpi only, whereas no difference occurred at the rest of the observation time points (Figure 2).

### 2.8. Suppressive Effect of OMW Against V. dahliae over Time

**Figure 3:**
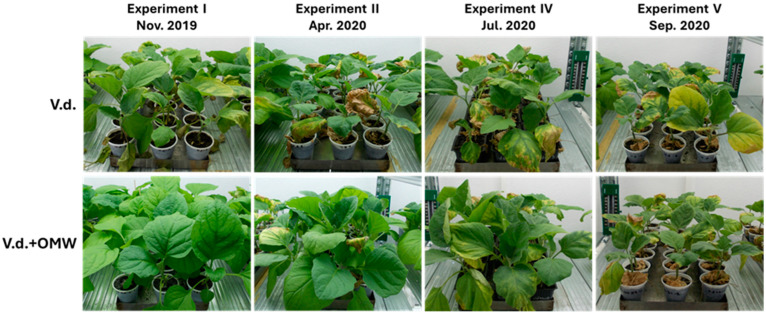
Disease symptoms on eggplants inoculated with *Verticillium dahliae*, previously treated with olive mill wastewater **(V.d. + OMW)** or not **(V.d.)**, in different experiments (I, II, IV and V) conducted at different time periods (November 2019, April 2020, July 2020 and September 2020) under the same conditions.

Data of disease severity on eggplants inoculated with high *V. dahliae* inoculum density (20 mL of 5 × 10^6^ conidia mL^−1^ suspension per plant) and treated with OMW (V.d. + OMW) or not (V.d.) across the different experiments (I, II, IV and V) conducted under the same conditions (in terms of plant host, fungal isolate, inoculum density, incubation conditions, etc.) but at different time periods are shown. The results are plotted together in Figure 2 and reveal that the suppressive effect of OMW is gradually decreased with time (Figure 3). In particular, OMW treatment in November 2019 (experiment I) eliminated verticillium wilt symptoms in eggplant; the disease progress was practically zero and the final disease severity did not exceed 4.06% at 30 dpi. Disease severity in OMW-treated plants was significantly lower compared to positive controls from 14 to 30 dpi. In April 2020 (experiment II), disease symptoms in OMW-treated plants progressed slowly, reaching a final disease severity of 32.91% at 32 dpi, and their disease severity was significantly lower compared to controls from 18 to 32 dpi. In July 2020 (experiment IV), OMW-treated plants showed a comparatively increased disease progress in relation to the previous experiments, finally reaching 56.78%, and their disease severity values were significantly lower than in controls from 18 to 32 dpi. Finally, in September 2020 (experiment V), disease progress in OMW-treated plants was further increased, reaching 70.16% at 32 dpi, while significant differences in disease severity between V.d + OMW-treated and V.d.-treated plants were not observed at most evaluation time points (Figure 2 and Figure 3). Interestingly, *V. dahliae* isolation ratio values in V.d. + OMW-treated plants were 0.10, 0.37, 0.48 and 0.86 in experiments I, II, IV and V, respectively (Table 2, Table 3, Table 5 and Table 6), suggesting that the gradual loss of OMW’s protective activity resulted in increased vascular colonization of plants by the pathogen over time.

### 2.9. Analysis of the Bacterial Microbial Diversity of the Tomato and Eggplant Plants

A total of 449.930 and 448.651 sequence read pairs were obtained from amplicon sequencing from the eggplant and tomato samples, respectively. After quality control, 359.600 and 350.924 high-quality amplicon sequences for tomato and eggplant samples were obtained, respectively, and they were used in the downstream analysis. Good’s coverage index verified that our sequencing effort provides adequate coverage of the microbial diversity (Supplementary Table S2).

**Figure 4:**
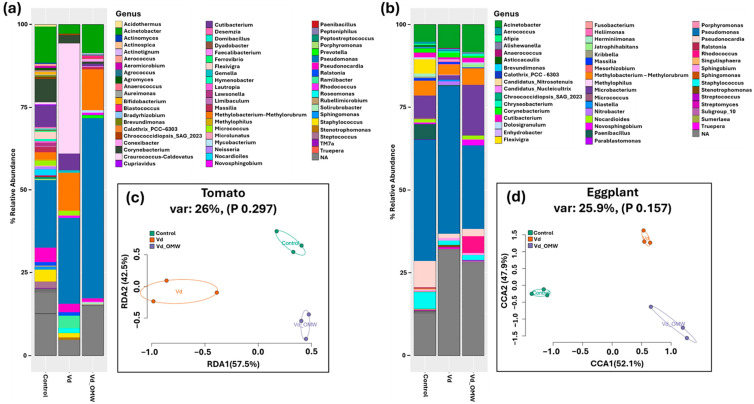
Stacked bar plots showing the relative abundance of the most dominant members of the bacterial community phylogenetically assigned to the genus levels in the tomato (**a**) and eggplant (**b**) plants, along with redundancy analysis (RDA) and canonical correspondence analysis (CCA) ordination plots of the bacterial community in tomato (**c**) and eggplant (**d**) plants, respectively. The relative abundance was calculated with the 200 most dominant ASVs in the dataset. Ellipses denote the grouping of the samples according to the treatment. Model parameters, variance and significance levels are provided in the panel above the graphs.

Firstly, we looked into the composition of the bacterial community in both tomato and eggplant plants of the study. In the case of both tomato and eggplant plants, approximately 50–60% of the relatively more abundant ASVs in all three treatments of the present study were classified in the genera of *Pseudomonas* (the most dominant), *Microbacterium* and *Methylobacterium-Methylorubrum* (Figure 4a,b). It is noteworthy that the genera *Craurococcus-Caldovatus* and *Ramlibacter* were observed in high abundance (almost 25%) only in the tomato plants treated with *V. dahliae* (Figure 4a). In contrast to tomato plants, in the eggplant plants, dominant taxa like *Flexivirga* and *Paenibacillus* were observed only in the control eggplant samples and not in the other treatments where *V. dahliae* was applied, while *Nitrobacter* ASVs seemed to be in low relative abundance only in the plants where *V. dahliae* was applied (Figure 4b).

Analysis of the β-diversity of bacteria showed that the samples were grouped according to the type of treatment employed in the three groups: (a) the control plants (control), (b) the plants treated with *V. dahliae* (Vd) and (c) the plants treated with *V. dahliae* and OMW (Vd_OMW) (Figure 4c,d). Despite their grouping, the bacterial communities in these three groups did not significantly differ (*p* > 0.05) in either plant species.

We further explored the impact of the different treatments on the α-diversity of bacteria in the tomato and eggplant plants. Significant differences between treatments were only evident in the tomato plants for the inverse Simpson index where the control samples carried significantly higher values compared to the *V. dahliae*-treated samples (Supplementary Table S3, Supplementary Figure S5A). On the contrary, we did not observe any statistically significant effects of the different treatments in the alpha-diversity of bacteria in the eggplant samples (Supplementary Table S3, Supplementary Figure S5b).

Finally, we looked for any specific patterns in the bacterial community showing clear association with the treatments employed. We identified ASV0020 belonging to the genus *Flexivigra* that showed significantly higher abundance in the tomato plants in the control treatment compared with the *V. dahliae*-treated samples (Supplementary Figure S6a), whereas no such patterns were evident in the eggplant samples.

### 2.10. Analysis of the Fungal Microbial Diversity of the Tomato and Eggplant Plants

After amplicon sequencing, a total of 429.382 and 384.565 sequence read pairs were obtained from the eggplant and tomato samples, respectively. Quality control reduced the number of sequences that were eventually used in the analysis by 13.2% for both tomato and eggplant plants. Good’s coverage index verified that our sequencing effort provides adequate coverage of the fungal diversity (Supplementary Table S4).

**Figure 5:**
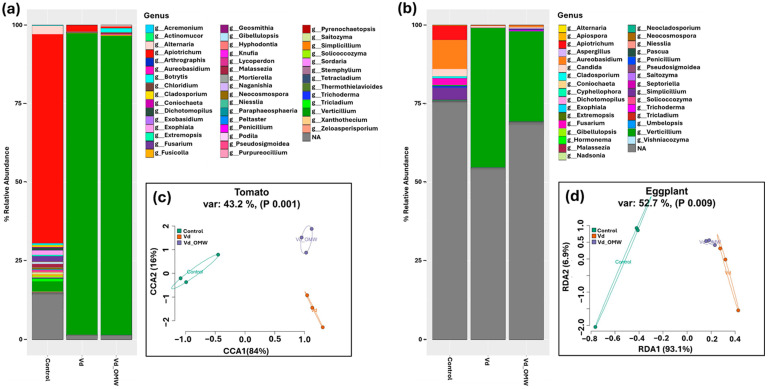
Stacked bar plots showing the relative abundance of the most dominant members of the fungal community phylogenetically assigned to the genus levels in the tomato (**a**) and eggplant (**b**) plants, along with redundancy analysis (RDA) and canonical correspondence analysis (CCA) ordination plots of the fungal community in tomato (**c**) and eggplant (**d**) plants, respectively. The relative abundance was calculated with the 200 most dominant ASVs in the dataset. Ellipses denote the grouping of the samples according to the treatment. Model parameters, variance and significance levels are provided in the panel above the graphs.

The phylogenetic composition of the fungal community in tomato plants varied greatly according to the treatment employed. Hence, a highly diverse community was evident in the control samples (not inoculated with *V. dahliae*) where the most dominant ASVs were associated with the genera *Apiotrichum*, *Alternaria* and *Verticillium* in the tomato plants (Figure 5a) and *Apiotrichum*, *Aureobasidium*, *Fusarium* and *Simplicilium* in the eggplants (Figure 5b). In contrast, the fungal community in the *V. dahliae*-inoculated samples in both plant species was dominated by ASVs assigned to the genus *Verticillium* (ca. 90% in tomato and 35–45% in the eggplant) (Figure 5a,b).

Analysis of the β-diversity of the fungal community in the tomato plants suggested a significant effect (*p* < 0.05) of the different treatments (explaining 43.2% of the derived variance) with samples from the different treatments forming three distinct groups based on the three different treatments (Figure 5c): (a) control plants, (b) plants treated with *V. dahliae* (Vd) and (c) plants treated with *V. dahliae* and OMW (Vd_OMW). This was not replicated in the eggplant plants, where the control plants formed a distinct cluster which statistically differed from the samples of the other two treatments (Vd and Vd_OMW) that formed a separate cluster (Figure 5d).

Regarding the effects of the treatments on the α-diversity of fungi, we noted significant effects only in the tomato plants (Supplementary Figure S7, Supplementary Table S5). Specifically, we observed significant values of Observed, Shannon and Pielou Evenness in the control samples compared to the samples treated with *V. dahliae* regardless of the further application of OMW or not.

Analysis of the differential abundance of fungal ASVs among the different treatments identified four ASVs belonging to *Apiotrichum* (ASV0003), *Exobasidium* (ASV00123), *Alternaria* (ASV0028) that were positively associated with the control tomato plants (Supplementary Figure S6b). On the other hand, in the eggplant samples, we identified five differentially abundant ASVs belonging to the genera *Candidata*, *Simplicillium* and *Cladosporium* that were positively associated with the control samples, and two ASVs belonging to *Verticillium* and *Nadsonia*, being positively associated with plants that have been inoculated with *V. dahliae* (Supplementary Figure S6c).

## 3. Discussion

Verticillium wilt caused by *V. dahliae* constitutes a serious threat for a broad range of economically important crops worldwide, since no effective chemical control is available. Therefore, alternative control strategies are intensively investigated. On the other hand, olive-producing countries face the challenge of managing olive mill waste (OMW) in an environmentally friendly and agronomically beneficial manner. Scientifically guided application of OMW to land has been proposed as a feasible way to successfully manage olive-derived wastes and plant diseases [4,10]. Herein, we tested whether soil application of raw OMW can protect vegetable crops against *V. dahliae* and investigated the potential disease-suppressive mechanisms.

Apart from the microsclerotial area, OMW reduced or nullified all fungal parameters evaluated, indicating a significant antifungal activity of OMW against *V. dahliae in vitro*. Correlation analyses revealed that the effects of OMW on growth rate, sporulation, spore germination and microsclerotium germination were dose-dependent. Such fungicidal activity of raw OMW, processed OMW (e.g., hydroxytyrosol-rich OMW, composted olive waste extracts) and individual components that are contained in OMW (e.g., the phenolic compounds oleuropein and hydroxytyrosol) against *V. dahliae* has been shown in several studies [5,9,15,16,17,18]. However, in all the above studies, *in vitro* inhibitory effects were assessed only on the mycelial growth and/or conidial germination of *V. dahliae*. In the present study, several microbial parameters were affected, with inhibition of microsclerotium germination being the most important. Microsclerotia constitute the primary inoculum structures in soil which germinate and infect host plants in nature [1]. Therefore, inhibition of microsclerotia germination due to OMW application in soil could lead to decreased disease incidence and thus, effective disease management in the field.

Root-drench application of OMW provided significant protection of eggplant and tomato against *V. dahliae* in planta. Likewise, a reduction in verticillium wilt symptom severity in horticultural crops and cotton by applying olive derivatives (like OMW composts, hydroxytyrosol-rich OMW and OMW-originating microorganisms) has also been reported [6,7,8,9,18,19]. However, the suppressive effect of raw OMW material against verticillium wilt in planta has never been shown previously. The aspect of direct application of raw OMW in the regions of origin is an important matter since the high regional distribution of olive mills in several countries like Greece could make the re-use of OMW completely unaffordable due to increased transportation and processing costs [20].

Τhe high OMW-conferred protection of eggplant in November 2019 gradually decreased in the following experiments conducted with the same OMW material, possibly due to the decreased phenolic content in OMW over time. Indeed, the total phenol content in non-sterilized OMW at the end (October 2020) was significantly lower than that measured at the beginning (July 2019) of the experiments. Feki et al. [21] also reported a remarkable decrease in concentrations of several phenolic compounds occurring in OMW after 5 months of storage, whereas hydroxytyrosol was the only compound that increased significantly in the meantime. A positive association between the total phenol content in composts and their suppressive effect against verticillium wilt in eggplant was also demonstrated by Markakis et al. [7].

Interestingly, re-isolations performed from OMW-treated plants inoculated with *V. dahliae* revealed several fungi (mostly *Trichoderma*-like strains) emerging from xylem chips solely or simultaneously with *V. dahliae*. Such fungal contaminants were not observed in *V. dahliae*-inoculated plants that had not been treated with OMW, indicating a differential texture of cultivable endophytic fungal inhabitants in OMW-treated plants compared to the non-treated ones. Although alterations in the structure of soil microbiome as a consequence of OMW application have been shown in the past [10,11,22], alterations in cultivable plant microbiome have never been reported previously.

Based on microbiome analysis, no significant differences were revealed among treatments that could be associated with disease suppressiveness. Some notable differences were identified between non-inoculated and *V. dahliae*-inoculated plants, irrespective of the treatment with OMW. This is the case of the bacterial ASV0020 belonging to the genus *Flexivigra* that was identified at significantly higher abundance in the control tomato plants compared to their *V. dahliae*-inoculated counterparts, suggesting a decreasing abundance pattern upon *V. dahliae* infection. Indeed, Snelders et al. [23] showed that *V. dahliae* secrets plant natriuretic peptide-like effectors that reduce the abundance of species of *Actinobacteria* (including *Flexivigra*), which otherwise reduces pathogen invasion. Likewise, fungal ASVs representing *Apiotrichum* (ASV0003), *Exobasidium* (ASV00123) and *Alternaria* (ASV0028) were found at higher abundance in control tomato plants compared to the *V. dahliae*-inoculated ones, suggesting a similar decreasing pattern as that mentioned above. In the case of eggplant, the abundance of the genera *Candidata* (ASV00030), *Simplicillium* (ASV00005) and *Cladosporium* (ASV00036) was significantly higher in the control plants, whereas *Nadsonia* (ASV00077) and *Verticillium* (ASV00002) were comparatively higher in plants that were inoculated with *V. dahliae*. The fungal genera *Alternaria*, *Cladosporium* and *Simplicillium* have been associated with plant welfare and have been pointed out either as potential biocontrol/plant growth-promoting agents or as indicators of asymptomatic status in plants [24,25,26]. Differential abundance analysis along with fungal re-isolation and qPCR revealed a significantly lower *V. dahliae* colonization in OMW-treated eggplants inoculated with the fungus compared to the non-treated ones. The observed lower fungal colonization was associated with decreased symptom severity.

Bioassays with sterilized OMW and/or soil suggest an unlikely association of soil- and OMW-originated microbiome with disease-suppressive mechanisms, since heat sterilization did not affect the protective activity of OMW in planta. This hypothesis is strengthened by the fact that the microbial strains isolated from OMW-treated soil and from asymptomatic OMW-treated plants could not provide sufficient protection of plants in pathogenicity assays. Contrarywise, other studies demonstrated that heat sterilization of olive derivatives resulted in complete or partial loss of their suppressive activity against *V. dahliae*, pointing out the microbial nature of their disease-suppressive mechanism [8,17]. Moreover, in contrast to studies that speculate the induction of a defence response mechanism in the plant upon OMW application [4,9], the non-significant protection of plants in split-root set-ups presented here indicates a non-systemic OMW-induced resistance mechanism in plants.

In view of all the above, data from the present study suggest that root-drench application of raw OMW can be an efficient practice to protect vegetable crops against *V. dahliae* and a sustainable way to manage OMW in olive-producing countries that have enacted specific legislation. It is evident that further study under field and controlled conditions is needed to optimize the beneficial effect of OMW and minimize the potential environmental risk, in order to utilize this valuable resource in modern cropping systems. Future research should address the suppressive activity of OMW against certain plant pathogens with respect to their composition, the olive mill system (two- and three-phase), the processed fruit (maturity, variety) and operating conditions. One step forward would be to elucidate the disease-suppressive mechanism and develop feasible delivery and application systems adapted to the crops of interest. The proper use of OMW (in terms of application method, dosage and timing) should be supported by scientific research focusing on the single-field level, considering the soil properties, cultivated species and phytopathological issues existing in individual fields.

## 4. Materials and Methods

### 4.1. Determination of Mineral Nutrients and Phenolic Content in Olive Mill Wastewater

Olive mill wastewater (OMW) was collected from a three-phase olive mill in the region of Tympaki, Heraklion, Greece, in spring 2019 and kept in an airtight plastic container at room temperature throughout the experiments.

Two samples of about 200 mL from each OMW treatment (sterilized by autoclaving at 121 °C for 30 min and non-sterilized OMW) that were used in the experiment were dried at 105 °C to constant weight, with weight measurements before and after drying used for calculation of the moisture percentage. A subsample of 0.1 g of the dried material was used to colorimetrically determine total N, after wet digestion [27]. Another subsample of 1 g was dried to ash at 550 °C and then solubilized with 5 mL of 20% HCl and diluted to 25 mL. The solution was then dispersed into 2 vessels, each one used for determination of the following: (a) Total concentration of P, K, Ca, Mg, Fe, Zn, Mn and Cu, using a dual view ICP-OES (Optima 8300, Perkin Elmer, Springfield, IL, USA). (b) Total B concentration, after the addition of an azomethin-H/EDTA reagent and colorimetric determination at 410 nm, using a visual spectrum photometer (PhotoLab 6100, Wissenschaftlich-Technische Werkstätten GmbH, Weilheim, Germany). Results of macronutrients were expressed as a percentage (%) of OMW fresh weight, whereas micronutrients were expressed as ppm of OMW fresh weight.

Total phenols were recovered by non-sterilized and sterilized OMW samples according to a previous method [7]. Each sample of sterile or non-sterile OMW (2 g) was mixed thoroughly with 40 mL of water in a falcon tube at 220 rpm for 12 h in a shaking device. The mixture was then centrifuged at 3500 rpm for 10 min, and the resulting aqueous phase was filtered directly in a 50 mL volumetric flask, which was subsequently filled to full volume with water. This procedure was repeated three times for each OMW sample. The total phenol content of each diluted filtrate was determined according to a method described by Markakis et al. [28]. In brief, an aliquot (1 mL) of the diluted phenol extract, 10 mL of an aqueous solution of Folin–Ciocalteau reagent (10% *v*/*v*) and 9 mL of an aqueous solution of sodium carbonate (7.5% *w*/*v*) were successively added to a screw-cup glass vial. The mixture was shaken well and remained in a dark place for 2 h. The absorbance was then measured at 765 nm, using a U-2900 Hitachi spectrophotometer (Tokyo, Japan). The total phenol content of each OMW sample, expressed in terms of gallic acid equivalent (g of gallic acid/L of OMW), was the mean of three measurements.

### 4.2. In Vitro Evaluation of Olive Mill Wastewater Against V. dahliae

The highly virulent *V. dahliae* isolate ‘999-1’ originating from a diseased eggplant was used in the following experiments [29]. The mycelial growth of *V. dahliae* was examined by transferring PDA discs of actively growing mycelium (5 mm in diameter) into the centre of new 92 mm diameter PDA plates (one disc per plate) containing either pure PDA (designated as V. d. + 0.00% OMW) or PDA supplemented with 0.01, 0.05, 0.1, 0.25, 0.50, 1.00 or 5.00% (*v*/*v*) sterilized OMW (V. d. + 0.01% OMW, V. d. + 0.05% OMW, V. d. + 0.1% OMW, V. d. + 0.25% OMW, V. d. + 0.5% OMW, V. d. + 1.00% OMW or V. d. + 5.00% OMW, respectively). Plates were incubated at 24 °C in the dark, and the colonies’ diameter was measured at 3, 7, 10 and 14 days post-inoculation (dpi). The growth rate of *V. dahliae* in different treatments was expressed in mm/day. At the end of the bioassays (14 dpi), the underside of the plates was scanned using a Samsung Xpress SL-M2875ND Laser Multifunction Printer (Samsung Electronics Co., Ltd., Suwon, Republic of Korea) at 1200 dots per inch, and the microsclerotial (black-pigmented) area on each plate image was determined manually using the image-processing software ImageJ 1.46r (National Institutes of Health, USA). Then, the microsclerotial area was estimated by measuring the number of pixels corresponding to this area and converting pixels to cm^2^. The number of spores was estimated by transferring a 5 mm diameter disc taken from the periphery of each culture into a 1.5 mL Eppendorf tube with 1 mL of sterilized distilled water, and vortexing vigorously for 30 s. The number of spores was measured with the use of a haematocytometer under a light microscope and expressed as the number of spores per 5 mm diameter disc. Moreover, actively growing mycelia from cultures’ periphery were prepared, and microscopic observations (30 readings per culture) were carried out to estimate hyphae width. Three replicated plates per OMW concentration were prepared and the overall experiment repeated twice (six replicated plates per OMW concentration in total).

To evaluate the effect of OMW on spore germination, conidial suspension was prepared as described previously for estimation of spore number, and the concentration was adjusted to 1 spore μL^−1^. Eighteen 1 μL volume drops were deposited onto PDA medium supplemented with OMW at the abovementioned concentrations (eighteen drops per plate). Plates were incubated at 24 °C in the dark for 3 days. Spore germination was assessed visually and estimated as a percentage of the emerging colonies. Likewise, to evaluate the effect of OMW on microsclerotium germination, microsclerotial suspension was prepared according to Markakis et al. [7]. In brief, microsclerotia were prepared by growing *V. dahliae* in sucrose sodium nitrate (SSN) liquid cultures in an orbital incubator for 3 weeks. Then, microsclerotia were centrifuged at 3000× *g* for 10 min at room temperature to remove the growth medium and air-dried in a laminar flow cabinet aseptically. Microsclerotia were resuspended in sterilized distilled water and their concentration was adjusted to 0.5 microslcerotia μL^−1^. Eighteen 2 μL volume drops were deposited onto PDA medium (eighteen drops per plate) supplemented with OMW at the abovementioned concentrations. Plates were incubated at 24 °C in the dark for 2 days. Microsclerotia were examined under a light microscope and microsclerotium germination was estimated as a percentage of the germinated microsclerotia. A microsclerotium was considered germinated when the germ tube was ≥ than the microsclerotium diameter. Three replicated plates per OMW concentration were prepared and the overall experiment repeated twice (six replicated plates per OMW concentration in total).

### 4.3. Plant Material

Plant material consisted of eggplant (cv Black Beauty) and tomato (hybrid Belladonna F1) seedlings at the one-true-leaf stage. Both genotypes are susceptible to *V. dahliae* [29]. To prepare seedlings, germinated seeds were sown in vermiculite, and when reaching the cotyledon stage, seedlings were transplanted in 100 mL capacity pots containing non-sterilized or heat-sterilized (autoclaved twice at 121 °C for 1 h) soil substrate (HuminSubstrat, Klasmann-3 Deilmann GmbH, Papenburg, Germany).

### 4.4. Optimal Dosage of OMW Application

Eggplant and tomato plants grown in 100 mL capacity pots were root-drenched with 0%, 5%, 10%, 25% or 50% (*v*/*v*) aqueous OMW solution (20 mL of each OMW solution per plant), and phytotoxicity was determined by assessing symptoms (i.e., leaf chlorosis, yellowing and wilting) and plant growth (plant height and fresh weight). It was revealed that 5% OMW did not cause phytotoxicity and thus was considered to be the optimal dosage for the following experiments.

### 4.5. OMW, Fungal Strains and V. dahliae Preparation for Pathogenicity Assays

Non-sterilized and heat-sterilized (autoclaved at 121 °C for 30 min) OMWs, as well as five fungal strains isolated from the disease-suppressive soil substrate (designated as KX1, KX2, KX3, KX6 and KX7) and one (KF8) isolated from an asymptomatic *V. dahliae*-inoculated eggplant in the experiment I, were evaluated in in planta bioassays. The six fungal strains and the highly virulent *V. dahliae* isolate 999-1 with proven pathogenicity on eggplant and tomato were prepared according to Markakis et al. [29]. In brief, conidia were produced by growing each fungus in potato dextrose broth (PDB) at 160 rpm and 24 °C in the dark for 5 days. Then, conidia were harvested by filtration through three layers of cheesecloth and the suspensions centrifuged at 3000× *g* for 10 min. *V. dahliae* spores were resuspended in sterilized distilled water and their concentration was adjusted to 5 × 10^6^ conidia mL^−1^ or 2 × 10^6^ conidia mL^−1^ for high or low inoculum density, respectively, whereas spores of the rest of the fungal strains were adjusted to 1 × 10^7^ conidia mL^−1^.

### 4.6. In Planta Bioassays

Plants at the one-true-leaf stage, grown in 100 mL capacity pots containing non-sterilized or heat-sterilized soil substrate (designated as ‘Steril. Soil’), were root-drenched with 20 mL of 5% non-sterilized OMW each (designated as ‘OMW’), with sterilized OMW (designated as ‘Steril. OMW’), or with 20 mL of 1 × 10^7^ conidia mL^−1^ suspension of each fungal strain individually (designated as ‘KX1’, ‘KX2’, ‘KX3’, ‘KX6’, ‘KX7’ or ‘KF8’) or mixed (‘MIXED’). The commercial biofungicide TRIANUM-P (Koppert B.V. Hellas) was applied at the appropriate dosage according to the manufacturer’s instructions (20 mL of 3 × 10^7^ cfu mL^−1^ per plant) and served as a *V. dahliae*-suppressive reference treatment in the present study (designated as ‘TRIANUM-P’). Plants that served as negative controls (no OMW/no fungal strain/no TRINANUM-P, designated as ‘Control-’) and those that served as positive controls (no OMW/no fungal strain/no TRIANUM-P/plus *V. dahliae*, designated as ‘V.d.’), were treated with 20 mL of water. One week later, plants (at the second-true-leaf stage) were inoculated with *V. dahliae* by drenching the soil substrate in each pot with 20 mL of a high (5 × 10^6^ conidia mL^−1^ suspension) (designated as ‘V.d. high’) or low (2 × 10^6^ conidia mL^−1^ suspension) (designated as ‘V.d. low’) inoculum density of *V. dahliae*. Plants that served as negative controls (control-) were treated with 20 mL of water. Plants were maintained under controlled conditions at 23 ± 2 °C with a 12 h light and dark cycle.

To evaluate the suppressive effect of OMW against *V. dahliae* in planta and investigate the potential disease-suppressive mechanisms, five independent experiments (experiments Ι, ΙΙ, III, IV and V) were conducted. In experiment Ι, bioassays with eggplant were set up in November 2019 and the following six treatments were applied: control-, V.d. high, V.d. low, V.d. high + OMW, V.d. low + OMW and V.d. high + TRIANUM-P. In experiment ΙΙ, bioassays with eggplant were set up in April 2020, and fourteen treatments were conducted: control-, V.d. high, V.d. low, V.d. high + OMW, V.d. high_Steril. soil + OMW, V.d. low + OMW, V.d. high + KX1, V.d. high + KX2, V.d. high + KX3, V.d. high + KX6, V.d. high + KX7, V.d. high + KF8, V.d. high + MIXED and V.d. high + TRIANUM-P. In experiment III, bioassays with tomato were set up in June 2020, and three treatments were included: control-, V.d., V.d. + OMW), with *V. dahliae* to be applied at the high inoculum density only (thereafter designated as V.d.). In experiment IV, bioassays with eggplant were set up in July 2020, and six treatments were applied: control-, V.d., V.d. + OMW, V.d. + Steril.OMW, V.d./-, V.d./OMW). In this experiment, apart from the typical single-pot set-ups (treatments: control-, V.d., V.d. + OMW and V.d. + Steril.OMW), split-root set-ups were also conducted according to Markakis et al. [30], to investigate direct and/or indirect effects of OMW on *V. dahliae* in planta. In the split-root set-ups, *V. dahliae* was applied to one side of the split-root system while the other side received water (treatment V.d./-), or the pathogen was applied to one side of the split-root system and the other side received OMW (treatment V.d./OMW). Finally, in experiment V, bioassays with eggplant were set up in September 2020, including three treatments: control-, V.d. and V.d. + OMW.

Within each experiment, each treatment consisted of 7 plants and each experiment was replicated three times (21 plants per treatment and experiment in total).

### 4.7. Disease Assessment

Verticillium wilt symptoms on eggplant were recorded at 2-, 3-, 4- or 7-day intervals from 8 to 35 days post-inoculation with *V. dahliae* (dpi) in different experiments. Bioassays were evaluated by estimating disease severity, disease incidence, mortality, and relative area under the disease progress curve (RAUDPC). Disease parameters were recorded according to Markakis et al. [7]. In brief, disease severity at each observation was calculated from the number of wilting leaves, as a percentage of a total number of leaves of each plant. Disease ratings were plotted over time to generate disease progress curves. Subsequently, the area under disease progress curve (AUDPC) was calculated by the trapezoidal integration method [31]. Disease was expressed as a percentage of the maximum possible area with reference to the maximum value potential reached over the whole period of each experiment and is referred to as relative AUDPC (RAUDPC). Disease incidence was estimated as the percentage of infected plants. Only plants with a final disease severity of ≥20% were considered infected, in order to discriminate between *V. dahliae*-associated disease symptoms and other weak symptoms occasionally observed. Mortality was estimated as the percentage of dead plants.

### 4.8. Plant Growth

Growth parameters were evaluated at the end of bioassays (at 30, 32, 35, 32 and 32 dpi for experiments Ι, II, III, IV and V, respectively). To estimate the effect of the abovementioned treatments on plant growth, all plants were clipped off at the soil surface level and their height, fresh weight and number of leaves were measured.

### 4.9. Isolation of Fungal Strains from Disease-Suppressive Soil and Asymptomatic Plants

Isolation of the fungal strains (encoded as KX1, KX2, KX3, KX6 and KX7) from disease-suppressive soil (treatment V.d. high + OMW in experiment I) was performed either directly by detaching the emerging fungal fructifications that developed on the soil surface and streaking them on APDA, or by macerating 100 g of soil in 300 mL sterilized distilled water, shaking at 100 rpm at 25 °C for 45 min and preparing 10-fold dilution series. In the latter case, 200 μL of each series was plated on APDA and incubated at 25 °C in the dark for 2 days. The plant-originated strain (code KF8) was isolated during pathogen re-isolation performed from an asymptomatic OMW-treated eggplant artificially infested with *V. dahliae*. Isolated fungal strains were sub-cultured onto new PDA plates to obtain pure fungal colonies and stored at 4 °C until use. Fungal strains were identified at the genus level (*Trichoderma* sp.) based on their morphological traits.

### 4.10. Verticillium Dahliae Re-Isolation

To verify the presence of the applied *V. dahliae* strain in plant tissues, the leaves of plants that had been cut above the soil level previously were removed and their stems were surface-disinfected by spraying with 95% ethyl alcohol and passing them quickly through a flame, thrice. For each plant, 3 xylem chips taken from different sites along the stem (base, middle and upper part of the stem) were aseptically placed onto acidified potato dextrose agar (PDA) after the removal of the phloem. Plates were then incubated at 24 °C in the dark for 14 days. The emerging fungi that grew out of tissue excisions were examined visually and under a light microscope and identified as *V. dahliae* according to their morphological characteristics [1]. Pathogen isolation ratio was expressed as the frequency of positive *V. dahliae* isolations of each plant.

### 4.11. DNA Extraction

To quantify *V*. *dahliae* DNA in vascular tissues of eggplant (treatments: control-, V.d. high and V.d. high + OMW in experiment II) and tomato plants (treatments: control-, V.d. and V.d. + OMW in experiment III), the stems of 21 plants per treatment and experiment were destructively sampled (7 composite samples consisting of 3 pooled stems each) by cutting to 2–3 mm long pieces after the removal of the phloem, and stored at −20 °C. Plant tissues were freeze-dried and ground to a fine powder by using an autoclaved mortar and pestle, in the presence of liquid nitrogen. Total DNA was isolated according to the Cetyltetramethyl ammonium bromide (CTAB) method [32] with slight modifications. Briefly, 100 mg of plant tissue was homogenized with the use of a mortar and pestle in the presence of liquid nitrogen. Tissue powder was transferred in a 1.5 mL Eppendorf tube, and 500 μL of 2× CTAB extraction buffer (100 mMTris-HCl, 20 mM EDTA, 1.4 M NaCl, 2% CTAB, 0.5% *v*/*v* β-mecraproethanol) was added and homogenized. The samples were incubated at 65 °C for 45 min with periodical vortexing and centrifuged at 10,000 rpm for 10 min. The supernatant (~250 μL) was transferred to new tubes, and equal amounts (~250 μL) of phenol/chloroform/isoamyl alcohol (25:24:1) were added and mixed by vortexing. Samples were centrifuged at 13,000 rpm for 15 min. The aqueous phase (~200 μL) was transferred into a new tube, and an equal amount of chilled isopropanol was added, followed by quick and gentle inversion, and incubated at −20 °C overnight. The DNA pellet was precipitated at 13,000 rpm for 20 min, washed with 500 μL of 70% ethanol and precipitated at 13,000 rpm for 5 min. The DNA pellet was then suspended in 40 μL of Tris-HCL (10 mM, pH = 8). Then, 2 μL RNAse A (5 mg mL^−1^) was added followed by incubation at 50 °C for 15 min. The purity and quantity of DNA were determined using a Q5000 UV-Vis Spectrophotometer (Quawell, San Jose, CA, USA). The final DNA concentration of each isolate was adjusted to 20 ng mL^−1^ and stored at −20 °C until use.

### 4.12. Verticillium Dahliae qPCR Quantification

Real-time quantitative PCR (qPCR) assays were conducted for the detection and quantification of *V. dahliae* DNA in plant tissues. The *V. dahliae ITS* region was amplified with the use of primers ITS1-F (5′-CCGCCGGTCCATCAGTCTCTCTGTTTATAC-3′) and ITS2-R (5′-CGCCTGCGGGACTCCGATGCGAGCTGTAAC-3′) [33]. The *actin* gene was used as an internal standard to normalize small differences in total DNA template amounts. The amplification of the *actin* gene was performed with the primer pair SolACT-F (5′-TTCCGTTGCCCAGAGGTCCT-3′) και SolACT-R (5′-TCGCCCTTTGAAATCCACATC-3′) [34]. All qPCR assays were carried out in a QuantStudio 3 Real-Time PCR System (ThermoFisher, Waltham, MA, USA) by using the PowerUp™ SYBR^®^ Green Master Mix kit (ThermoFisher, Waltham, MA, USA). The qPCR performance included an initial denaturation at 95 °C for 3 min; followed by 40 cycles of 30 s of denaturation at 95 °C, 30 s of annealing at 60 °C, and 30 s of extension at 72 °C; and a final extension step at 60 °C for 1 min. The relative DNA quantity of *V. dahliae* was determined by using the 2^−ΔΔCT^ method [35]. The real-time qPCR reactions were performed in duplicate, and the absence of nonspecific products and primer dimers was confirmed by the analysis of melting curves. For data analysis, average threshold cycle (Ct) values were calculated for each gene of interest on the basis of seven independent biological samples (seven composite samples consisting of 3 pooled plants each).

### 4.13. Analysis of the Microbial Community Composition of the Eggplant and Tomato Plants

Apart from *V. dahliae* qPCR quantification, DNA samples extracted from eggplant (experiment II) and tomato (experiment III) plants were employed for microbiome analysis. DNA quality was then assessed via electrophoresis on 0.8% agarose gels, while the DNA concentration was determined via fluorometer measurement with Qubit v.2 Bacterial and fungal community changes in the plants were tracked using multiplex amplicon sequencing, following our internal protocol [36]. Sequencing was performed by Admera Health (South Plainfield, NJ, USA) on an Illumina NovaSeq platform (San Diego, CA, USA), employing the SP sequencing kit to generate 250 bp paired-end reads. The V4 region of the bacterial 16S rRNA gene was amplified with primers 515f–806r [37], and the ITS2 region of fungi was amplified using primers fITS7–ITS4 [38]. Thermal cycling conditions and primer details are provided in Supplementary Table S7.

After the acquisition of the NGS data, a pre-analysis of the sequences was performed by de-multiplexing the samples using the Flexbar programme, version 3.0.3 [39]. Quality control and chimera identification of the sequences were then conducted, followed by error correction in the DNA sequences and assembly of the sequencing inserts from the two reads of each insert to form amplicon sequence variants (ASVs) using the dada2 package [40] in R software version 4.2.2 (R Core Team, 2020). The sequence data of the V4 region of the bacteria and the ITS2 region of fungi were deposited in NCBI GenBank under the BioProject number PRJNA1227913.

Phylogenetic classification was carried out by comparing each ASV with reference sequences from databases. In this case, the Silva SSU v138 database [41] formatted for dada2 was used for bacterial ASV classification, and the Unite version 9 database [42] was used for fungal ASVs. Using the phyloseq v1.38.0 package in R, which allows the creation of objects containing a range of information such as the ASV read count table, DNA sequences of the ASVs (via the Biostrings v2.62.0 package), the taxonomic classification table, and the experimental design information for the samples [43], data management was performed during the subsequent statistical analysis.

In this data analysis, we initially calculated alpha- and beta-diversity indices like Shannon, inverse Simpson, Observed Richness, Pielou, low abundance, and the Good’s coverage index for the prokaryotic and fungal communities in tomato and eggplant plants using the phyloseq v1.38.0 package and the entropart v1.6.10 package [44]. Between samples, statistical differences were determined via ANOVA or its non-parametric counterpart, the Kruskal–Wallis test, using the agricolae v1.3.5 package [45].

Further, the effects of the different treatments on the beta-diversity of the plant bacterial and fungal communities were analyzed via canonical correspondence analysis (CCA) [46] or redundancy analysis (RDA) [47], depending on the range of values on the first axis of the Detrended Correspondence Analysis (DCA) [48], and Permutational Analysis of Variance (PERMANOVA) [49] using the Adonis version 0.0.1 package [50].

### 4.14. Statistics

Analysis of variance (ANOVA) was employed to determine the effects of replication (1, 2 or 3), treatment (control-, V.d. high, V.d. low, V.d. high + OMW, V.d. low + OMW and V.d. high + TRIANUM-P in experiment I, control-, V.d. high, V.d. low, V.d. high + OMW, V.d. high_Steril. soil + OMW, V.d. low + OMW, V.d. high + KX1, V.d. high + KX2, V.d. high + KX3, V.d. high + KX6, V.d. high + KX7, V.d. high + KF8, V.d. high + MIXED, V.d. high + TRIANUM-P in experiment II, control-, V.d. and V.d. + OMW in experiment III, control-, V.d., V.d. + OMW, V.d. + Steril.OMW, V.d./-, V.d./OMW in experiment IV, and control-, V.d. and V.d. + OMW in experiment V), and their interaction on disease incidence (DI), final disease severity (FDS), mortality (M), relative area under disease progress curve (relative AUDPC), isolation ratio (IR) and *V. dahliae* DNA quantity (qPCR), and on leaf number (L), plant height (H) and plant fresh weight (FW) (Supplementary Table S6). Prior to ANOVA, homogeneity of variance across treatments was evaluated, and an arcsine transformation was applied to normalize variance. When a significant *F* test was obtained for treatments (*p* ≤ 0.05), the data were subjected to means separation by Tukey’s honestly significant difference test. Morphological and physiological characteristics of *V. dahliae* in *in vitro* assays were also analyzed by Tukey’s test (*p* ≤ 0.05). Moreover, standard errors of means were calculated. Associations among different fungal parameters (growth rate, sporulation, hyphae width, microslerotial area, spore germination, microsclerotium germination) and OMW content in PDA plates were checked by determining the Pearson’s r correlation coefficients (*p* ≤ 0.05).

## Figures and Tables

**Table 1:** Values (±standard errors) of fungal parameters of *Verticillium dahliae* grown on PDA amended with sterilized olive mill wastewater (OMW) at various concentrations (0.00%, 0.001%, 0.05%, 0.10%, 0.25%, 0.50%, 1.00% and 5.00%).

Treatment	Fungal Parameters ^a^					
	Growth Rate (mm/day) ^b^	Spores (×10^5^) per 5 mm Diameter Disc ^c^	Hyphae Width (μm) ^d^	Microsclerotial Area (cm^2^) ^e^	Spore Germination (%) ^f^	Microsclerotium Germination (%) ^g^
V. d. + 0.00% OMW	3.84 ± 0.18 a	27.92 ± 7.87 ab	3.35 ± 0.13 a	12.72 ± 2.09 a	63.66 ± 4.28 a	37.03 ± 4.97 a
V. d. + 0.01% OMW	3.46 ± 0.22 ab	21.08 ± 2.16 abcd	2.58 ± 0.13 b	8.39 ± 2.31 a	59.03 ± 2.70 ab	26.88 ± 5.92 ab
V. d. + 0.05% OMW	3.24 ± 0.23 abc	38.00 ± 5.18 a	2.17 ± 0.11 bc	7.67 ± 2.31 a	56.01 ± 4.22 abc	23.14 ± 5.37 ab
V. d. + 0.10% OMW	3.11 ± 0.21 abc	25.42 ± 4.97 abc	1.78 ± 0.08 cd	4.93 ± 1.70 a	50.23 ± 2.20 abc	28.19 ± 3.60 ab
V. d. + 0.25% OMW	2.95 ± 0.24 bc	11.83 ± 2.07 bcd	2.02 ± 0.09 cd	5.59 ± 1.98 a	54.40 ± 3.80 abc	24.56 ± 3.96 ab
V. d. + 0.50% OMW	2.66 ± 0.10 bc	10.25 ± 1.25 cd	1.86 ± 0.08 cd	5.14 ± 1.28 a	47.92 ± 2.50 bc	20.52 ± 4.47 abc
V. d. + 1.00% OMW	2.61 ± 0.12 c	8.92 ± 0.97 cd	1.72 ± 0.08 d	5.09 ± 1.28 a	43.98 ± 2.82 c	10.94 ± 5.11 bc
V. d. + 5.00% OMW	2.45 ± 0.08 c	6.17 ± 0.95 d	2.07 ± 0.11 cd	7.93 ± 0.38 a	0.00 ± 0.00 d	5.89 ± 2.82 c

^a^ Each value represents the mean of 6 replicates. Within columns, values followed by the same letter are not significantly different according to Tukey’s HSD test at *p* ≤ 0.05. ^b^ Isolates were grown on PDA amended with sterilized olive mill wastewater at various concentrations at 24 °C in the dark and their colonies’ diameter was measured on days 3, 7, 10 and 14. ^c^ At the end of the bioassays (14 dpi), a 5 mm diameter disc taken from the periphery of each culture was transferred into a 1.5 mL Eppendorf tube containing 1 mL of sterilized distilled water and vortexed for 30 s. The number of spores was measured using a haematocytometer under a light microscope. ^d^ Mycelia from cultures’ periphery were prepared and microscopic observations (30 readings per culture) were carried out to estimate hyphae width. ^e^ At the end of the bioassays (14 dpi), the underside of the plates was scanned using a Samsung Xpress SL-M2875ND Laser Multifunction Printer (Samsung Electronics Co., Ltd., Suwon, Republic of Korea), and the microsclerotial (black) area on each plate image was determined using the image-processing software ImageJ 1.46r (National Institutes of Health, United States). ^f^ Conidial suspension was adjusted to 1 spore μL^−1^, eighteen 1 μL volume drops were deposited onto PDA medium (six replicated PDA plates per treatment) and the plates were incubated at 24 °C in the dark for 3 days. Spore germination was assessed visually and estimated as a percentage of the emerging colonies. ^g^ Microsclerotial suspension was adjusted to 0.5 microsclerotia μL^−1^ and eighteen 2 μL volume drops were deposited onto PDA medium in plates that were incubated at 24 °C in the dark for 2 days. Microsclerotia were examined under a light microscope and microsclerotium germination was estimated as a percentage of the germinated microsclerotia. A microsclerotium was considered germinated when the germ tube was ≥ the microsclerotium diameter.

**Table 2:** Values (±standard errors) of disease and growth parameters for eggplants mock-inoculated (control-) or artificially inoculated with high or low inoculum density of *Verticillium dahliae* and treated with olive mill wastewater (OMW) or TRIANUM-P or non-treated, in experiment Ι.

Treatment	Disease Parameters ^a^					Plant Growth Parameters ^b^		
	DI (%)	FDS (%)	M (%)	RAUDPC (%)	IR (0–1)	L	H (cm)	FW (gr)
Control-	0.00 ± 0.00 c	0.00 ± 0.00 c	0.00 ± 0.00 b	0.00 ± 0.00 c	0.00 ± 0.00 c	6.76 ± 0.14 a	18.31 ± 0.34 a	6.56 ± 0.17 a
V.d. high	90.48 ± 6.15 a	68.49 ± 5.43 a	47.62 ± 9.91 a	26.76 ± 2.95 a	0.59 ± 0.08 a	6.24 ± 0.14 ab	17.00 ± 0.39 ab	2.89 ± 0.31 b
V.d. low	52.38 ± 6.14 b	34.27 ± 7.91 b	9.52 ± 6.15 b	6.80 ± 2.01 bc	0.29 ± 0.14 b	6.52 ± 0.13 ab	18.29 ± 0.39 a	6.19 ± 0.49 a
V.d. high + OMW	14.29 ± 14.29 c	4.06 ± 2.36 c	0.00 ± 0.00 b	0.25 ± 0.15 c	0.10 ± 0.06 bc	6.25 ± 0.14 ab	16.75 ± 0.34 ab	6.47 ± 0.37 a
V.d. low + OMW	4.76 ± 4.76 c	1.90 ± 1.90 c	0.00 ± 0.00 b	0.66 ± 0.66 c	0.13 ± 0.07 bc	6.05 ± 0.13 b	17.71 ± 0.48 ab	5.96 ± 0.37 a
V.d. high + TRIANUM-P	52.38 ± 6.74 b	33.45 ± 7.44 b	4.76 ± 4.76 b	7.88 ± 1.94 b	0.25 ± 0.16 bc	6.48 ± 0.11 ab	16.14 ± 0.56 b	5.87 ± 0.42 a

^a^ Disease parameters were evaluated periodically on the basis of external symptoms during a period of 30 days after root drenching (dpi) with 20 mL of *V. dahliae* conidial suspension per plant at high (5 × 10^6^ conidia mL^−1^) or low (2 × 10^6^ conidia mL^−1^) inoculum density. One week prior to inoculation with *V. dahliae*, plants were root-drenched with OMW (20 mL of 5% OMW per plant). TRIANUM-P was also applied by root drenching (20 mL of 3 × 10^7^ cfu mL^−1^ per plant). DI = final disease incidence; FDS = final disease severity; M = mortality; RAUDPC = relative area under the disease progress curve with reference to the maximum value potentially reached over each assessment period; IR = isolation ratio. Each value represents the mean of 21 plants. Within columns, values followed by the same letter are not significantly different according to Tukey’s HSD test at *p* ≤ 0.05. ^b^ Plant growth parameters were evaluated at 30 dpi. L = number of leaves; H = plant height; FW = plant fresh weight. Each value represents the mean of 21 plants. Within columns, values followed by the same letter are not significantly different according to Tukey’s HSD test at *p* ≤ 0.05.

**Table 3:** Values (±standard errors) of disease and growth parameters for eggplants mock-inoculated (control-) or artificially inoculated with high or low inoculum density of *Verticillium dahliae* and treated with olive mill wastewater (OMW) in non-sterilized or sterilized soil, various microbial strains (KX1, KX2, KX3, KX6, KX7 or KF8), TRIANUM-P or non-treated (control-), in experiment ΙI.

Treatment	Disease Parameters ^a^						Plant Growth Parameters ^b^		
	DI (%)	FDS (%)	M (%)	RAUDPC (%)	IR (0–1)	qPCR	L	H (cm)	FW (gr)
Control-	0.00 ± 0.00 e	0.00 ± 0.00 c	0.00 ± 0.00 b	0.00 ± 0.00 e	0.00 ± 0.00 d	0.00 ± 0.00 b	7.10 ± 0.41 a	15.22 ± 0.84 ab	7.73 ± 0.47 a
V.d. high	100.00 ± 0.00 a	75.22 ± 1.57 a	33.33 ± 10.29 ab	38.40 ± 1.48 a	0.98 ± 0.02 a	3.90 ± 1.10 a	6.95 ± 0.26 a	11.57 ± 0.37 c	2.53 ± 0.16 c
V.d. low	85.72 ± 6.73 ab	66.81 ± 5.06 a	23.81 ± 14.02 ab	29.63 ± 3.28 ab	0.84 ± 0.06 a	ne	7.05 ± 0.29 a	13.21 ± 0.48 bc	4.24 ± 0.55 bc
V.d. high + OMW	42.86 ± 14.02 c	32.91 ± 7.73 b	9.52 ± 6.15 ab	12.84 ± 3.37 cd	0.37 ± 0.09 c	1.12 ± 0.35 b	6.90 ± 0.15 a	15.12 ± 0.62 ab	6.26 ± 0.62 ab
V.d. high_Steril. soil + OMW	57.14 ± 15.79 bc	35.96 ± 7.20 b	0.00 ± 0.00 b	10.23 ± 2.74 de	0.49 ± 0.10 bc	ne	7.10 ± 0.23 a	16.74 ± 0.65 a	6.27 ± 0.53 ab
V.d. low + OMW	42.86 ± 11.98 c	28.34 ± 7.55 b	14.28 ± 6.73 ab	8.20 ± 2.78 de	0.30 ± 0.09 cd	ne	6.90 ± 0.15 a	15.14 ± 0.57 ab	6.12 ± 0.61 ab
V.d. high + KX1	100.00 ± 0.00 a	72.80 ± 1.16 a	19.05 ± 9.91 ab	30.81 ± 1.92 ab	ne	ne	6.62 ± 0.15 a	12.21 ± 0.41 c	2.78 ± 0.28 c
V.d. high + KX2	90.48 ± 6.15 ab	69.54 ± 5.30 a	52.38 ± 12.30 a	30.29 ± 3.09 ab	ne	ne	6.62 ± 0.33 a	13.38 ± 0.31 bc	3.00 ± 0.45 c
V.d. high + KX3	100.00 ± 0.00 a	70.68 ± 2.40 a	23.81 ± 9.52 ab	31.78 ± 2.17 ab	ne	ne	7.10 ± 0.28 a	13.00 ± 0.43 bc	3.29 ± 0.25 c
V.d. high + KX6	100.00 ± 0.00 a	76.15 ± 1.35 a	42.86 ± 9.52 ab	33.15 ± 1.57 ab	ne	ne	6.48 ± 0.13 a	12.81 ± 0.41 bc	2.52 ± 0.14 c
V.d. high + KX7	100.00 ± 0.00 a	75.28 ± 2.32 a	42.86 ± 9.52 ab	32.19 ± 2.08 ab	ne	ne	6.52 ± 0.11 a	12.17 ± 0.27 bcd	2.80 ± 0.27 c
V.d. high + KF8	95.24 ± 4.76 ab	70.16 ± 3.37 a	33.33 ± 10.29 ab	26.55 ± 2.60 ab	ne	ne	6.71 ± 0.18 a	14.05 ± 0.59 bcd	3.74 ± 0.45 c
V.d. high + MIXED	85.71 ± 9.91 ab	63.32 ± 6.26 a	23.76 ± 9.52 ab	28.28 ± 3.53 ab	ne	ne	6.62 ± 0.41 a	12.26 ± 0.54 c	3.48 ± 0.51 c
V.d. high + TRIANUM-P	80.95 ± 9.91 abc	61.58 ± 7.00 a	37.95 ± 13.48 ab	22.91 ± 3.37 bc	0.76 ± 0.08 ab	0.56 ± 0.09 b	6.57 ± 0.44 a	12.67 ± 0.69 bc	3.76 ± 0.59 c

^a^ Disease parameters were evaluated periodically on the basis of external symptoms during a period of 32 days after root drenching (dpi) with 20 mL of *V. dahliae* conidial suspension per plant at high (5 × 10^6^ conidia mL^−1^) or low (2 × 10^6^ conidia mL^−1^) inoculum density. One week prior to inoculation with *V. dahliae*, plants transplanted in non-sterilized or sterilized soil were root-drenched with OMW (20 mL of 5% OMW per plant). Separated or mixed microbial strains (KX1, KX2, KX3, KX6, KX7, KF8 or MIXED) were also applied by root drenching 20 mL of 1 × 10^7^ spores mL^−1^ per plant, whereas TRIANUM-P was applied by root drenching 20 mL of 3 × 10^7^ cfu mL^−1^ per plant. DI = final disease incidence; FDS = final disease severity; M = mortality; RAUDPC = relative area under the disease progress curve with reference to the maximum value potentially reached over each assessment period; IR = isolation ratio. ‘ne’ indicates that IR and qPCR were not estimated for the respective treatments. Each value represents the mean of 21 plants. Within columns, values followed by the same letter are not significantly different according to Tukey’s HSD test at *p* ≤ 0.05. ^b^ Plant growth parameters were evaluated at 32 dpi. L = number of leaves; H = plant height; FW = plant fresh weight. Each value represents the mean of 21 plants. Within columns, values followed by the same letter are not significantly different according to Tukey’s HSD test at *p* ≤ 0.05.

**Table 4:** Values (±standard errors) of disease and growth parameters for tomato plants mock-inoculated (control-) or artificially inoculated with high inoculum density of *Verticillium dahliae* and treated with olive mill wastewater (OMW) or non-treated, in experiment ΙII.

Treatment	Disease Parameters ^a^						Plant Growth Parameters ^b^		
	DI (%)	FDS (%)	M (%)	RAUDPC (%)	IR (0–1)	qPCR	L	H (cm)	FW (gr)
Control-	0.00 ± 0.00 c	0.00 ± 0.00 c	0.00 ± 0.00 a	0.00 ± 0.00 c	0.00 ± 0.00 c	0.002 ± 0.001 c	7.71 ± 0.10 a	38.39 ± 0.78 a	11.26 ± 0.34 a
V.d.	95.27 ± 4.76 a	42.76 ± 1.89 a	0.00 ± 0.00 a	25.14 ± 1.43 a	0.62 ± 0.07 a	0.182 ± 0.026 a	7.67 ± 0.13 a	34.74 ± 0.83 b	8.98 ± 0.23 c
V.d. + OMW	52.38 ± 9.91 b	22.36 ± 4.87 b	0.00 ± 0.00 a	11.54 ± 2.62 b	0.38 ± 0.09 b	0.097 ± 0.021 b	7.50 ± 0.11 a	36.13 ± 0.75 ab	10.21 ± 0.37 b

^a^ Disease parameters were evaluated periodically on the basis of external symptoms during a period of 35 days after root drenching (dpi) with 20 mL of *Verticillium dahliae* conidial suspension per plant at high (5 × 10^6^ conidia mL^−1^) inoculum density. One week prior to inoculation with *V. dahliae*, plants were root-drenched with OMW (20 mL of 5% OMW per plant). DI = final disease incidence; FDS = final disease severity; M = mortality; RAUDPC = relative area under the disease progress curve with reference to the maximum value potentially reached over each assessment period; IR = isolation ratio. Each value represents the mean of 21 plants. Within columns, values followed by the same letter are not significantly different according to Tukey’s HSD test at *p* ≤ 0.05. ^b^ Plant growth parameters were evaluated at 35 dpi. L = number of leaves; H = plant height; FW = plant fresh weight. Each value represents the mean of 21 plants. Within columns, values followed by the same letter are not significantly different according to Tukey’s HSD test at *p* ≤ 0.05.

**Table 5:** Values (±standard errors) of disease and growth parameters for eggplants mock-inoculated (control-) or artificially inoculated with high inoculum density of *Verticillium dahliae* and treated with sterilized or non-sterilized olive mill wastewater (OMW) or non-treated, and for eggplants in a split-root set-up with half of the split-root system being inoculated with the fungus and the other half with water (V.d./-) or half of the split-root system being inoculated with the fungus and the other half being treated with olive mill wastewater (V.d./OMW), in experiment ΙV.

Treatment	Disease Parameters ^a^					Plant Growth Parameters ^b^		
	DI (%)	FDS (%)	M (%)	RAUDPC (%)	IR (0–1)	L	H (cm)	FW (gr)
Control-	0.00 ± 0.00 c	0.00 ± 0.00 c	0.00 ± 0.00 b	0.00 ± 0.00 c	0.00 ± 0.00 b	7.57 ± 0.22 ab	17.79 ± 0.72 a	10.67 ± 0.54 a
V.d.	100.00 ± 0.00 a	79.91 ± 1.64 a	57.14 ± 14.02 a	37.24 ± 1.39 a	0.78 ± 0.06 a	6.62 ± 0.22 b	13.67 ± 0.52 b	2.93 ± 0.18 c
V.d. + OMW	80.95 ± 6.73 ab	56.78 ± 6.51 b	23.81 ± 6.15 ab	23.72 ± 3.03 b	0.48 ± 0.11 ab	6.95 ± 0.30 ab	16.31 ± 0.68 a	5.01 ± 0.71 bc
V.d. + Steril.OMW	76.19 ± 9.52 b	53.32 ± 7.08 b	14.28 ± 6.73 b	21.77 ± 3.12 b	0.63 ± 0.09 a	7.29 ± 0.27 ab	15.83 ± 0.65 ab	5.50 ± 0.75 bc
V.d./-	85.72 ± 6.73 ab	58.54 ± 6.50 ab	14.28 ± 6.73 b	28.15 ± 4.05 ab	0.70 ± 0.10 a	7.57 ± 0.21 ab	18.05 ± 0.60 a	8.48 ± 1.43 ab
V.d./OMW	85.72 ± 6.73 ab	62.13 ± 5.83 ab	14.29 ± 9.91 b	28.71 ± 3.11 ab	0.74 ± 0.09 a	7.71 ± 0.23 a	16.86 ± 0.70 a	7.60 ± 1.25 ab

^a^ Disease parameters were evaluated periodically on the basis of external symptoms during a period of 32 days after root drenching (dpi) with 20 mL of *V. dahliae* conidial suspension per plant at high (5 × 10^6^ conidia mL^−1^) inoculum density. One week prior to inoculation with *V. dahliae*, plants were root-drenched with non-sterilized or sterilized OMW (20 mL of 5% OMW per plant). DI = final disease incidence; FDS = final disease severity; M = mortality; RAUDPC = relative area under the disease progress curve with reference to the maximum value potentially reached over each assessment period; IR = isolation ratio. Each value represents the mean of 21 plants. Within columns, values followed by the same letter are not significantly different according to Tukey’s HSD test at *p* ≤ 0.05. ^b^ Plant growth parameters were evaluated at 32 dpi. L = number of leaves; H = plant height; FW = plant fresh weight. Each value represents the mean of 21 plants. Within columns, values followed by the same letter are not significantly different according to Tukey’s HSD test at *p* ≤ 0.05.

**Table 6:** Values (±standard errors) of disease and growth parameters for eggplants mock-inoculated (control-) or artificially inoculated with high inoculum density of *Verticillium dahliae* and treated with olive mill wastewater (OMW) or non-treated, in experiment V.

Treatment	Disease Parameters ^a^					Plant Growth Parameters ^b^		
	DI (%)	FDS (%)	M (%)	RAUDPC (%)	IR (0–1)	L	H (cm)	FW (gr)
Control-	0.00 ± 0.00 b	0.00 ± 0.00 b	0.00 ± 0.00 a	0.00 ± 0.00 b	0.00 ± 0.00 b	7.29 ± 0.22 a	15.65 ± 0.40 a	7.87 ± 0.32 a
V.d.	100.00 ± 0.00 a	70.35 ± 1.97 a	28.57 ± 11.34 a	41.37 ± 1.71 a	0.92 ± 0.04 a	6.19 ± 0.15 b	11.86 ± 0.36 b	2.46 ± 0.26 b
V.d. + OMW	100.00 ± 0.00 a	70.16 ± 1.41 a	19.05 ± 9.91 a	36.62 ± 1.49 b	0.86 ± 0.06 a	6.19 ± 0.13 b	11.79 ± 0.27 b	2.26 ± 0.11 b

^a^ Disease parameters were evaluated periodically on the basis of external symptoms during a period of 32 days after root drenching (dpi) with 20 mL of *Verticillium dahliae* conidial suspension per plant at high (5 × 10^6^ conidia mL^−1^) inoculum density. One week prior to inoculation with *V. dahliae*, plants were root-drenched with OMW (20 mL of 5% OMW per plant). DI = final disease incidence; FDS = final disease severity; M = mortality; RAUDPC = relative area under the disease progress curve with reference to the maximum value potentially reached over each assessment period; IR = isolation ratio. Each value represents the mean of 21 plants. Within columns, values followed by the same letter are not significantly different according to Tukey’s HSD test at *p* ≤ 0.05. ^b^ Plant growth parameters were evaluated at 32 dpi. L = number of leaves; H = plant height; FW = plant fresh weight. Each value represents the mean of 21 plants. Within columns, values followed by the same letter are not significantly different according to Tukey’s HSD test at *p* ≤ 0.05.

## Data Availability

The data that support the findings of the present study are available from the authors upon reasonable request.

## Supplementary Materials

The following supporting information can be downloaded at https://www.mdpi.com/article/10.3390/plants14060867/s1: Figure S1: Verticillium wilt disease severity on eggplant mock-inoculated (control-) or inoculated with 20 mL of high (5 × 10^6^ conidia mL^−1^) or low (2 × 10^6^ conidia mL^−1^) inoculum density of *Verticillium dahliae*, treated with olive mill wastewater (OMW) or TRIANUM-P or non-treated at 12, 14, 16, 19, 23, 26, 28 and 30 days post-inoculation (experiment I). Figure S2: Verticillium wilt disease severity on eggplant mock-inoculated (control-) or inoculated with 20 mL of high (5 × 10^6^ conidia mL^−1^) or low (2 × 10^6^ conidia mL^−1^) inoculum density of *Verticillium dahliae*, treated with olive mill wastewater (OMW), separated or mixed microbial strains (KX1, KX2, KX3, KX6, KX7, KF8 or MIXED), TRIANUM-P or non-treated controls at 11, 18, 25, and 32 days post-inoculation (experiment II). Plants transplanted in sterilized soil and treated with OMW were also inoculated with high inoculum density (V.d. high_Steril. soil + OMW). Figure S3: Verticillium wilt disease severity on tomato mock-inoculated (control-) or inoculated with 20 mL of high (5 × 10^6^ conidia mL^−1^) inoculum density of *Verticillium dahliae*, treated with olive mill wastewater (OMW) or non-treated at 11, 14, 18, 21, 25, 28, 32 and 35 days post-inoculation (experiment III). Figure S4: Verticillium wilt disease severity index on eggplant mock-inoculated (control-) or inoculated with 20 mL of high (5 × 10^6^ conidia mL^−1^) inoculum density of *Verticillium dahliae*, treated with sterilized or non-sterilized olive mill wastewater (OMW) and on eggplant in a split-root set-up with the half of the split-root system being inoculated with the fungus and the other half with water (V.d./-) or the half of the split-root system being inoculated with the fungus and the other half being treated with olive mill wastewater (V.d./OMW), at 8, 11, 14, 18, 21, 25, 28 and 32 days post-inoculation (experiment IV). Figure S5: Temporal patterns of the α-diversity indices of the bacterial communities in the tomato (a) and eggplant (b) plants of the study. Figure S6: Heatmap showing the dominant differentially abundant ASVs of the bacteria community in the tomato plants (a), and the fungal community in the tomato (b) and eggplant (c) plants of the study. Figure S7: Temporal patterns of the α-diversity indices of the fungal communities in the tomato (a) and eggplant (b) plants of the study. Table S1: Chemical analysis of non-sterilized and heat-sterilized olive mill wastewater (OMW) used in the study at the beginning (July 2019) and the end (October 2020) of the experiments. Table S2: The sequence numbers per sample for the bacteria, obtained and remaining from the various quality control steps, and the successful coverage of the sample by the resulting high-quality sequences. Table S3: Per-sample α-diversity indices for the bacterial microbial communities for both plants of the study. Table S4: The sequence numbers per sample for the fungi, obtained and remaining from the various quality control steps, and the successful coverage of the sample by the resulting high-quality sequences. Table S5: Per-sample α-diversity indices for the fungal microbial communities for both plants of the study. Table S6: Analysis of variance (ANOVA) for disease and plant growth parameters on eggplant and tomato plants artificially inoculated with *Verticillium dahliae* and treated with various applications, in five different experiments (Ι, II, III, IV and V). Table S7: Primers, sequences and thermocycling conditions used to amplify the 16S rRNA and ITS2 genes for bacteria and fungi, respectively.

## Abbreviations

The following abbreviations are used in this manuscript:

OMW	Olive mill wastewater
GR	Growth rate
MA	Microsclerotial area
SG	Spore germination
MG	Microsclerotium germination
ASV	Amplicon sequence variant
NGS	Next-generation sequencing
CCA	Canonical correspondence analysis
RDA	Redundancy analysis
DCA	Detrended Correspondence Analysis
PERMANOVA	Permutational Analysis of Variance
