# Supplementary material for: Biocontrol Potential of Raw Olive Mill Waste Against Verticillium dahliae in Vegetable Crops
Source: Plants (Basel). 2025 Mar 10;14(6):867. doi: 10.3390/plants14060867 (PMC11944966; doi:10.3390/plants14060867)
Supplement: Supplementary file 1 [file plants-14-00867-s001.zip › Supplementary Figures/Supplementary Figure S1.pdf]

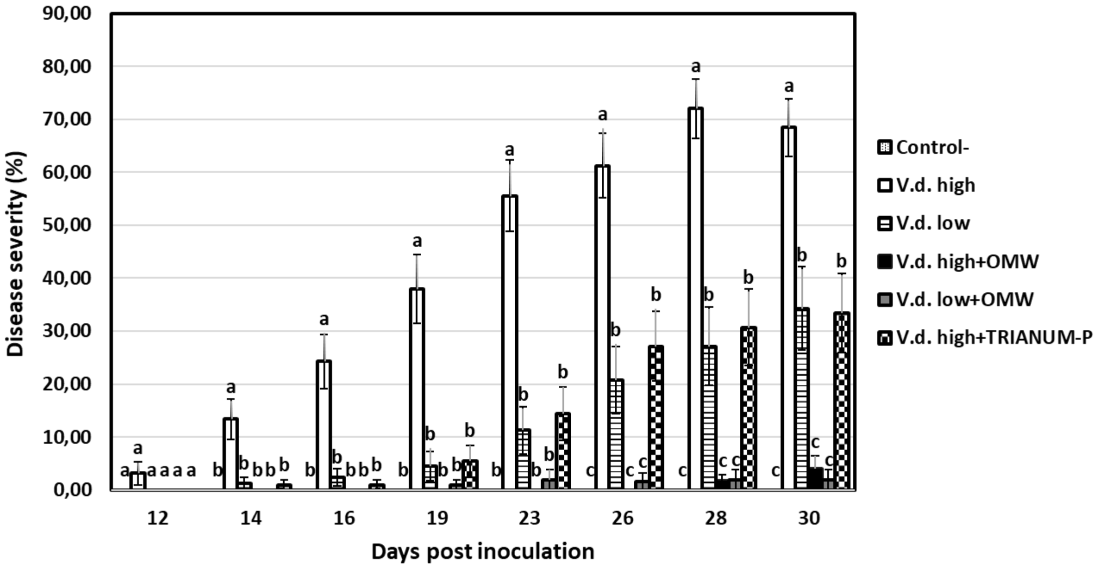

Figure S1. Verticillium wilt disease severity on eggplant mock inoculated (control-) or inoculated with 20 ml of high (5 × 10^6 conidia ml^{-1}) or low (2 × 10^6 conidia ml^{-1}) inoculum density of *Verticillium dahliae*, treated with olive mill wastewater (OMW) or TRIANUM-P or non treated at 12, 14, 16, 19, 23, 26, 28 and 30 days post inoculation (experiment I). Each column represents the mean of 21 plants. Columns at each observation time point followed by the same letter are not significantly different according to Tukey's HSD test at $P \leq 0.05$. Vertical bars indicate standard errors.
