# Supplementary material for: Biocontrol Potential of Raw Olive Mill Waste Against Verticillium dahliae in Vegetable Crops
Source: Plants (Basel). 2025 Mar 10;14(6):867. doi: 10.3390/plants14060867 (PMC11944966; doi:10.3390/plants14060867)
Supplement: Supplementary file 1 [file plants-14-00867-s001.zip › Supplementary Figures/Supplementary Figure S3.pdf]

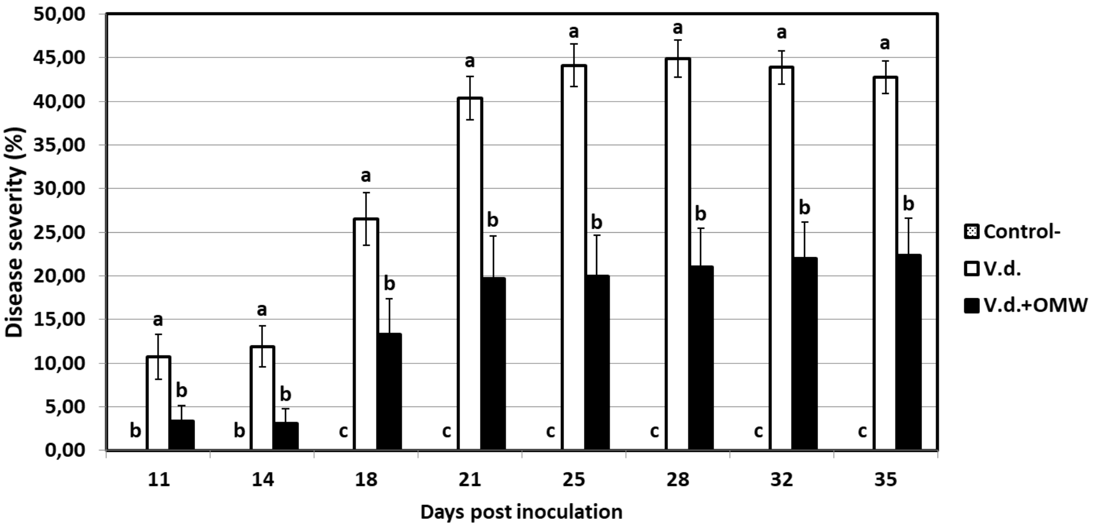

Figure S3. Verticillium wilt disease severity on tomato mock inoculated (control-) or inoculated with 20 ml of high (5×10^6 conidia ml^{-1}) inoculum density of *Verticillium dahliae,* treated with olive mill wastewater (OMW) or non treated at 11, 14, 18, 21, 25, 28, 32 and 35 days post inoculation (experiment III). Each column represents the mean of 21 plants. Columns at each observation time point followed by the same letter are not significantly different according to Tukey's HSD test at $P \leq 0.05$. Vertical bars indicate standard errors.
