# Supplementary material for: Biocontrol Potential of Raw Olive Mill Waste Against Verticillium dahliae in Vegetable Crops
Source: Plants (Basel). 2025 Mar 10;14(6):867. doi: 10.3390/plants14060867 (PMC11944966; doi:10.3390/plants14060867)
Supplement: Supplementary file 1 [file plants-14-00867-s001.zip › Supplementary Figures/Supplementary Figure S4.pdf]

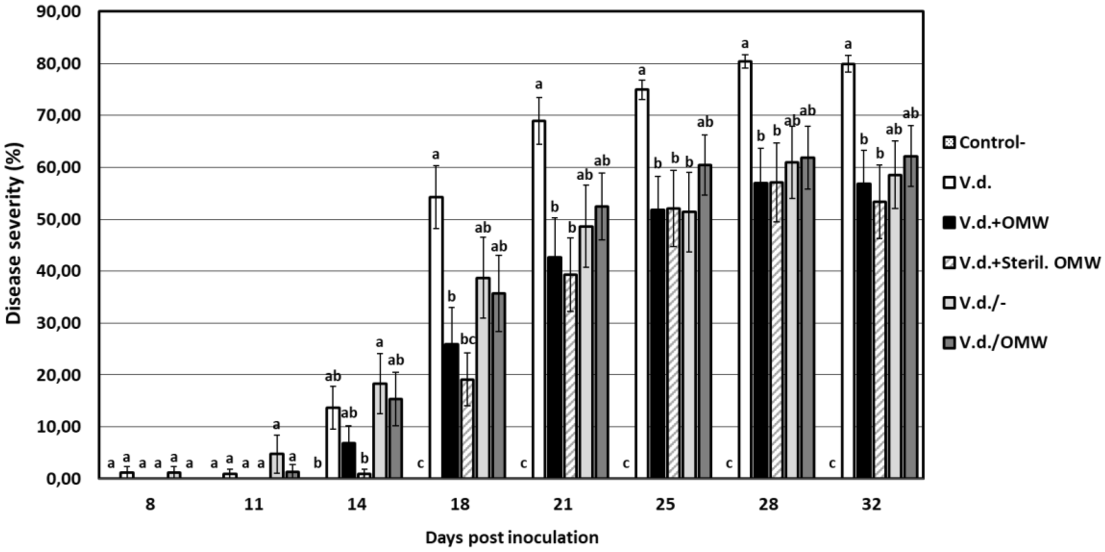

Figure S4. Verticillium wilt disease severity index on eggplant mock inoculated (control-) or inoculated with 20 ml of high (5×10^6 conidia ml^{-1}) inoculum density of *Verticillium dahliae*, treated with sterilized or non-sterilized olive mill wastewater (OMW) and on eggplant in a split-root set-up with the half of the split-root system being inoculated with the fungus and the other half with water (V.d./-) or the half of the split-root system being inoculated with the fungus and the other half being treated with olive mill wastewater (V.d./OMW), at 8, 11, 14, 18, 21, 25, 28 and 32 days post inoculation (experiment IV). Each column represents the mean of 21 plants. Columns at each observation time point followed by the same letter are not significantly different according to Tukey's HSD test at $P \leq 0.05$. Vertical bars indicate standard errors.
