# Supplementary material for: Biocontrol Potential of Raw Olive Mill Waste Against Verticillium dahliae in Vegetable Crops
Source: Plants (Basel). 2025 Mar 10;14(6):867. doi: 10.3390/plants14060867 (PMC11944966; doi:10.3390/plants14060867)
Supplement: Supplementary file 1 [file plants-14-00867-s001.zip › Supplementary Figures/Supplementary Figure S6_new.pdf]

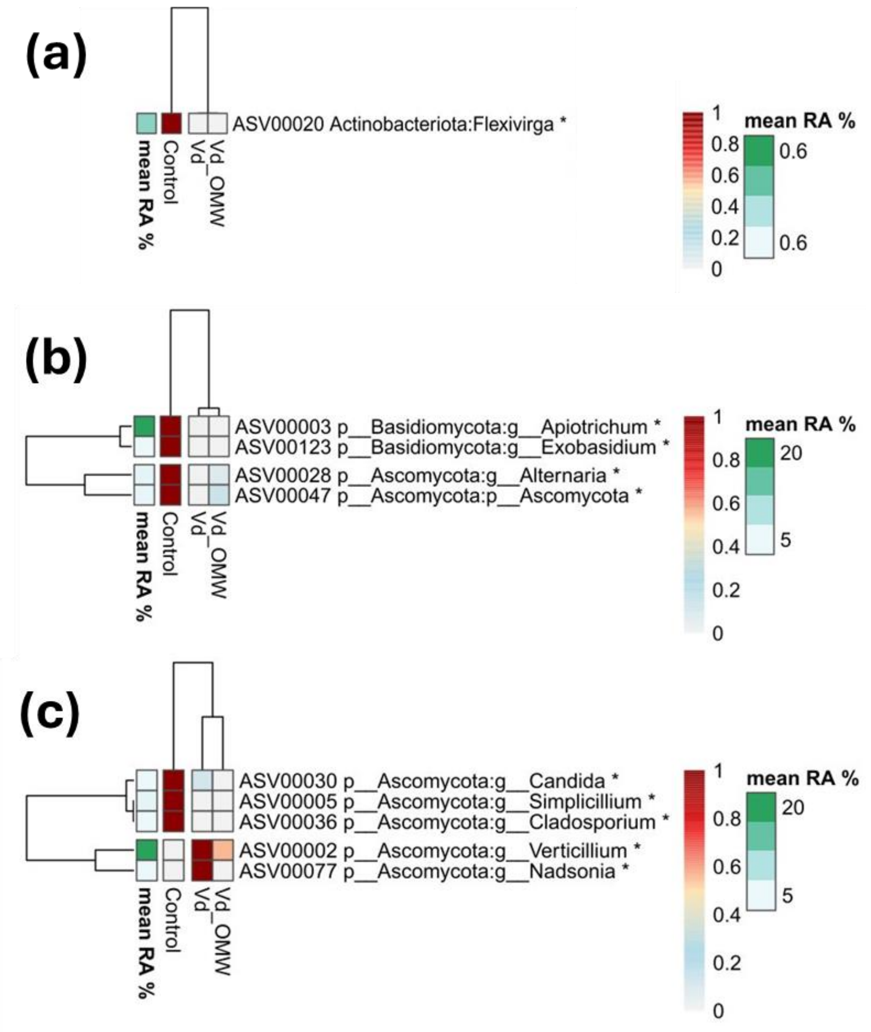

Figure S6. Heatmap showing the dominant differentially abundant ASVs of the bacteria community in the tomato plants **(a)**, and the fungal community in the tomato **(b)** and eggplant **(c)** plants of the study. Hierarchical clustering dendrograms are presented for all the samples and the ASVs (relative abundance differences between samples for each ASV are normalized as a 0 to 1 range for lowest to highest values; provided as white to grey squares according to the key), while the overall mean dataset relative abundance of each ASV is provided in the white to green column and key. The taxonomy affiliation is provided for each ASV, while the stars next to the ASV numbers show the between group significance. α cutoff notation: '***' P = 0.001, '**' P = 0.01, '*' P = 0.05 '.' P = 0.1, ' ' P = 1.
