# Supplementary material for: Biocontrol Potential of Raw Olive Mill Waste Against Verticillium dahliae in Vegetable Crops
Source: Plants (Basel). 2025 Mar 10;14(6):867. doi: 10.3390/plants14060867 (PMC11944966; doi:10.3390/plants14060867)
Supplement: Supplementary file 1 [file plants-14-00867-s001.zip › Supplementary Figures/Supplementary Figure S7_new.pdf]

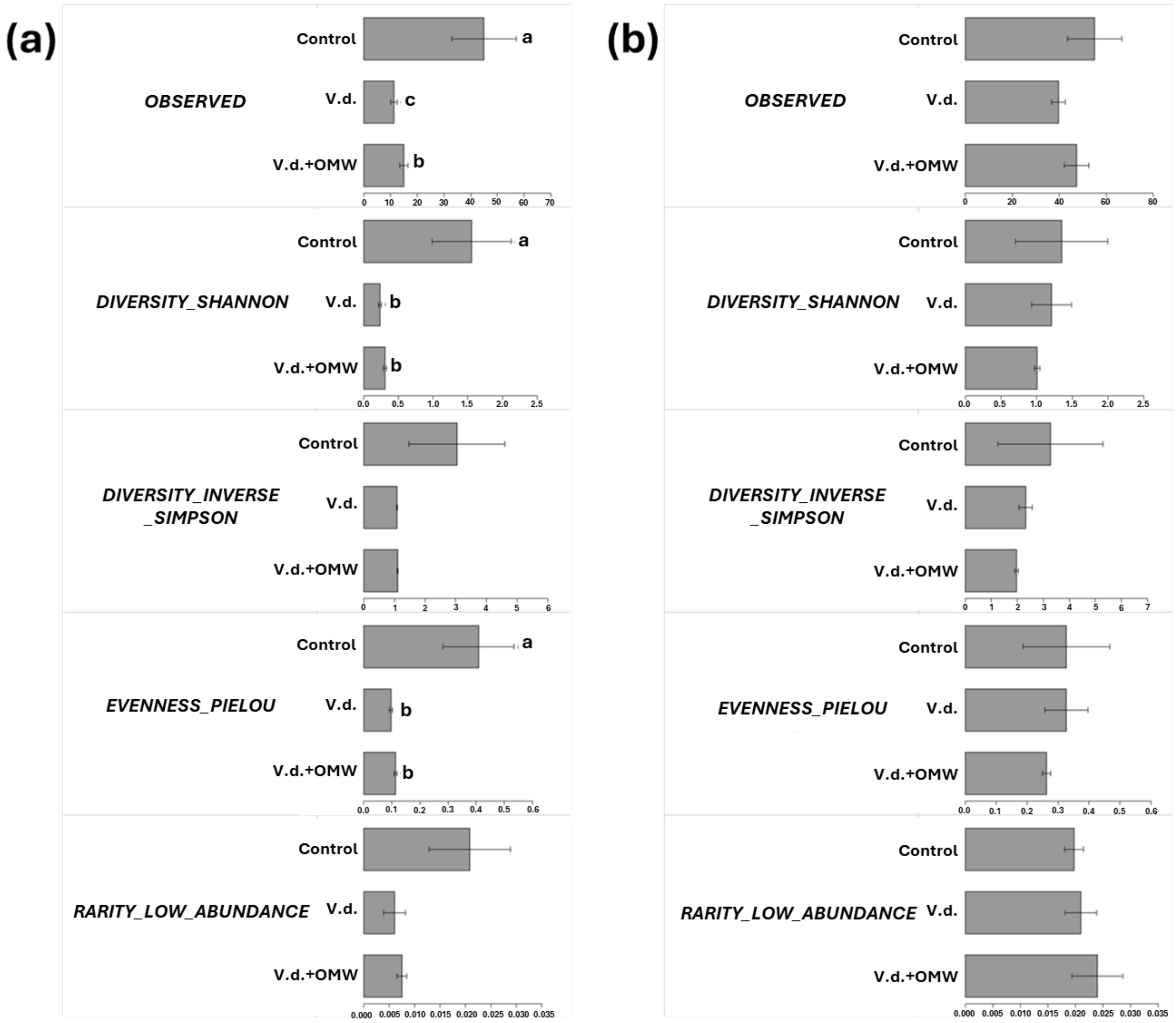

Figure S7. Temporal patterns of the α-diversity indices of the fungal communities in the tomato **(a)** and eggplant **(b)** plants of the study.
