# Supplementary material for: Biocontrol Potential of Raw Olive Mill Waste Against Verticillium dahliae in Vegetable Crops
Source: Plants (Basel). 2025 Mar 10;14(6):867. doi: 10.3390/plants14060867 (PMC11944966; doi:10.3390/plants14060867)
Supplement: Supplementary file 1 [file plants-14-00867-s001.zip › Supplementary Tables/Supplementary Table S1.pdf]

Table S1. Chemical analysis of non-sterilized and heat-sterilized olive mill wastewater (OMW) used in the study at the beginning (July 2019) and the end (October 2020) of the experiments

**Properties**[a]	**Non-sterilized OMW (July 2019)**	**Sterilized OMW (July 2019)**	**Non-sterilized OMW (October 2020)**[b]
Moisture (%)	91.050	91.110	ne
N (% FW)	0.145	0.200	ne
P (% FW)	0.040	0.057	ne
K (% FW)	0.654	0.564	ne
Ca (% FW)	0.121	0.172	ne
Mg (% FW)	0.042	0.037	ne
Fe (ppm FW)	47.721	58.710	ne
Zn (ppm FW)	6.730	8.108	ne
Mn (ppm FW)	3.822	5.654	ne
Cu (ppm FW)	3.195	3.600	ne
B (ppm FW)	4.940	2.227	ne
Total phenols (g gallic acid / L OMW)	7.707±0.168 a	6.494±0.044 b	6.003±0.042 c

[a] For moisture, macro- and micro- nutrients values represent the mean of 2 measurements whereas for total phenols values represent the mean of 3 measurements per sample. Values (± standard errors) of total phenols that are not followed by the same letter are significantly different according to Tukey's HSD test at $P \leq 0.05$.

[b] 'ne' indicates that macro- and micro- nutrients were not estimated in non-sterilized OMW in October 2020.
