# Supplementary material for: Biocontrol Potential of Raw Olive Mill Waste Against Verticillium dahliae in Vegetable Crops
Source: Plants (Basel). 2025 Mar 10;14(6):867. doi: 10.3390/plants14060867 (PMC11944966; doi:10.3390/plants14060867)
Supplement: Supplementary file 1 [file plants-14-00867-s001.zip › Supplementary Tables/Supplementary Table S2.pdf]

Table S2. The sequence numbers per sample for the bacteria, obtained and remaining from the various quality control steps, and the successful coverage of the sample by the resulting high-quality sequences

			Sequence Number		
**Plant**	**Treatment**	**Replications**	**Raw**	**Entered statistical analysis**	**Good's coverage**
Eggplant	Control	1	22202	18422	1,000
Eggplant	Control	2	99248	78658	1,000
Eggplant	Control	3	98802	77989	1,000
Eggplant	Vd	1	103854	83456	1,000
Eggplant	Vd	2	52200	40825	1,000
Eggplant	Vd	3	9359	7821	1,000
Eggplant	Vd_OMW	1	31803	26415	1,000
Eggplant	Vd_OMW	2	9419	7740	1,000
Eggplant	Vd_OMW	3	23043	18274	1,000
Tomato	Control	1	67393	52786	1,000
Tomato	Control	2	78202	60455	1,000
Tomato	Control	3	110161	87040	1,000
Tomato	Vd	1	6983	5591	1,000
Tomato	Vd	2	234	172	1,000
Tomato	Vd	3	54552	43553	1,000
Tomato	Vd_OMW	1	6016	4850	1,000
Tomato	Vd_OMW	2	3954	3188	1,000
Tomato	Vd_OMW	3	121156	93289	1,000
