# Supplementary material for: Biocontrol Potential of Raw Olive Mill Waste Against Verticillium dahliae in Vegetable Crops
Source: Plants (Basel). 2025 Mar 10;14(6):867. doi: 10.3390/plants14060867 (PMC11944966; doi:10.3390/plants14060867)
Supplement: Supplementary file 1 [file plants-14-00867-s001.zip › Supplementary Tables/Supplementary Table S3.pdf]

Table S3. Per-sample α-diversity indices for the bacterial microbial communities for both plants of the study

Plant	Treatment	Replicates	shannon	inverse_simpson	pielou	rarity_low_abundance
Eggplant	Control	1	6,630	329,556	0,888	0,581
Eggplant	Control	2	6,626	322,131	0,886	0,592
Eggplant	Control	3	6,495	296,696	0,889	0,577
Eggplant	Vd	1	5,861	269,549	0,940	0,272
Eggplant	Vd	2	6,903	434,423	0,899	0,667
Eggplant	Vd	3	6,741	437,584	0,916	0,679
Eggplant	Vd_OMW	1	6,537	361,343	0,932	0,707
Eggplant	Vd_OMW	2	6,469	337,154	0,928	0,689
Eggplant	Vd_OMW	3	5,589	158,675	0,924	0,219
Tomato	Control	1	6,288	279,003	0,887	0,507
Tomato	Control	2	6,174	269,209	0,898	0,503
Tomato	Control	3	6,264	288,039	0,895	0,514
Tomato	Vd	1	6,107	246,052	0,891	0,466
Tomato	Vd	2	5,768	199,538	0,904	0,316
Tomato	Vd	3	5,917	212,659	0,898	0,409
Tomato	Vd_OMW	1	6,161	200,389	0,852	0,450
Tomato	Vd_OMW	2	6,113	212,075	0,863	0,452
Tomato	Vd_OMW	3	6,218	223,074	0,854	0,450
