# Supplementary material for: Biocontrol Potential of Raw Olive Mill Waste Against Verticillium dahliae in Vegetable Crops
Source: Plants (Basel). 2025 Mar 10;14(6):867. doi: 10.3390/plants14060867 (PMC11944966; doi:10.3390/plants14060867)
Supplement: Supplementary file 1 [file plants-14-00867-s001.zip › Supplementary Tables/Supplementary Table S4.pdf]

Table S4. The sequence numbers per sample for the fungi, obtained and remaining from the various quality control steps, and the successful coverage of the sample by the resulting high-quality sequences

			Sequence Numbers		
**Plant**	**Treatment**	**Replications**	**Raw**	**Entered statistical analysis**	**Good's coverage**
Eggplant	Control	1	15522	14013	1,00000
Eggplant	Control	2	44408	40290	1,00000
Eggplant	Control	3	38425	34008	1,00000
Eggplant	Vd	1	17011	14161	1,00000
Eggplant	Vd	2	30754	26128	1,00000
Eggplant	Vd	3	65529	55587	1,00000
Eggplant	Vd_OMW	1	93322	80717	0,99999
Eggplant	Vd_OMW	2	40549	35087	1,00000
Eggplant	Vd_OMW	3	39045	33431	1,00000
Tomato	Control	1	74782	65958	1,00000
Tomato	Control	2	31793	27540	1,00000
Tomato	Control	3	47716	41789	1,00000
Tomato	Vd	1	22295	19236	1,00000
Tomato	Vd	2	44148	38144	1,00000
Tomato	Vd	3	56754	49017	1,00000
Tomato	Vd_OMW	1	73266	63222	1,00000
Tomato	Vd_OMW	2	38224	33138	1,00000
Tomato	Vd_OMW	3	40404	34646	1,00000
