# Supplementary material for: Biocontrol Potential of Raw Olive Mill Waste Against Verticillium dahliae in Vegetable Crops
Source: Plants (Basel). 2025 Mar 10;14(6):867. doi: 10.3390/plants14060867 (PMC11944966; doi:10.3390/plants14060867)
Supplement: Supplementary file 1 [file plants-14-00867-s001.zip › Supplementary Tables/Supplementary Table S5.pdf]

Table S5. Per-sample α-diversity indices for the fungal microbial communities for both plants of the study

Plant	Treatment	Replicates	shannon	inverse_simpson	pielou	rarity_low_abundance
Eggplant	Control	1	0,737	1,279	0,196	0,019
Eggplant	Control	2	0,675	1,241	0,178	0,017
Eggplant	Control	3	2,640	7,308	0,606	0,023
Eggplant	Vd	1	1,761	2,810	0,463	0,017
Eggplant	Vd	2	1,008	2,177	0,283	0,027
Eggplant	Vd	3	0,859	1,982	0,234	0,019
Eggplant	Vd_OMW	1	0,953	1,851	0,237	0,033
Eggplant	Vd_OMW	2	0,991	1,966	0,273	0,017
Eggplant	Vd_OMW	3	1,077	2,091	0,278	0,022
Tomato	Control	1	0,909	1,453	0,299	0,005
Tomato	Control	2	2,687	6,157	0,659	0,032
Tomato	Control	3	1,067	1,499	0,266	0,025
Tomato	Vd	1	0,195	1,067	0,089	0,003
Tomato	Vd	2	0,265	1,102	0,107	0,006
Tomato	Vd	3	0,245	1,091	0,096	0,010
Tomato	Vd_OMW	1	0,342	1,124	0,118	0,007
Tomato	Vd_OMW	2	0,263	1,085	0,102	0,006
Tomato	Vd_OMW	3	0,313	1,107	0,119	0,009
