# Supplementary material for: Biocontrol Potential of Raw Olive Mill Waste Against Verticillium dahliae in Vegetable Crops
Source: Plants (Basel). 2025 Mar 10;14(6):867. doi: 10.3390/plants14060867 (PMC11944966; doi:10.3390/plants14060867)
Supplement: Supplementary file 1 [file plants-14-00867-s001.zip › Supplementary Tables/Supplementary Table S6.pdf]

Table S6. Analysis of variance (ANOVA) for disease and plant growth parameters on eggplant and tomato plants artificially inoculated with *Verticillium dahliae* and treated with various applications, in five different experiments (I, II, III, IV and V)

		***F* values**[a]								
		**Disease parameters**						**Plant growth parameters**		
		**Experiment I (Eggplant -*V. dahliae* bioassays)**								
**Source**	**df**[b]	**Disease incidence**	**Final disease severity**	**Mortality**	**Relative AUDPC**	**Isolation ratio**	**qPCR**	**Leaf number**	**Plant height**	**Plant fresh weight**
Replication	2	0.68	0.85	1.05	1.74	1.86	-	0.74	1.69	0.31
Treatment	5	18.71***	26.56***	11.64***	37.51***	9.37***	-	3.61**	4.20**	13.39***
Replication × Treatment	10	0.86	0.47	0.59	0.96	0.24	-	0.53	0.31	0.90
		**Experiment II (Eggplant -*V. dahliae* bioassays)**								
**Source**	**df**[b]	**Disease incidence**	**Final disease severity**	**Mortality**	**Relative AUDPC**	**Isolation ratio**	**qPCR**	**Leaf number**	**Plant height**	**Plant fresh weight**
Replication	2	1.58	0.85	1.16	0.65	1.42	(2)1.95	0.26	1.28	0.75
Treatment	13	12.98***	21.61***	2.95**	18.69***	22.16***	(3)14.58***	0.72	8.36***	13.94***
Replication × Treatment	26	0.82	0.62	1.48	0.67	0.65	(6)2.18	0.07	1.71*	0.82
		**Experiment III (Tomato -*V. dahliae* bioassays)**								
**Source**	**df**[b]	**Disease incidence**	**Final disease severity**	**Mortality**	**Relative AUDPC**	**Isolation ratio**	**qPCR**	**Leaf number**	**Plant height**	**Plant fresh weight**
Replication	2	0.97	1.07	-	1.23	0.92	2.09	0.12	2.87	2.40
Treatment	2	65.70***	66.98***	-	52.97***	22.17***	26.21**	1.02	8.75**	13.74***
Replication × Treatment	4	2.00	1.94	-	0.76	0.23	2.21	1.23	1.93	1.04
		**Experiment IV (Eggplant -*V. dahliae* bioassays)**								
**Source**	**df**[b]	**Disease incidence**	**Final disease severity**	**Mortality**	**Relative AUDPC**	**Isolation ratio**	**qPCR**	**Leaf number**	**Plant height**	**Plant fresh weight**
Replication	2	2.11	0.35	0.41	0.39	0.46	-	1.38	0.90	0.15
Treatment	5	53.29***	25.65***	4.22**	21.10***	4.52**	-	2.94*	6.10***	9.07***
Replication × Treatment	10	3.06*	1.17	0.96	1.60	0.71	-	0.76	1.19	1.03
		**Experiment V (Eggplant -*V. dahliae* bioassays)**								
**Source**	**df**[b]	**Disease incidence**	**Final disease severity**	**Mortality**	**Relative AUDPC**	**Isolation ratio**	**qPCR**	**Leaf number**	**Plant height**	**Plant fresh weight**
Replication	2	0.01	0.44	1.48	0.55	0.34	-	0.52	0.12	0.33
Treatment	2	668.50***	847.11***	2.04	291.72***	150.24***	-	14.43***	36.68***	174.09***
Replication × Treatment	4	0.01	1.45	0.43	0.83	0.55	-	2.03	0.23	1.03

[a] Symbols '*', '**' and '***' indicate significance at $P \leq 0.05$, 0.01 and 0.001 levels, respectively, according to the *F* test.
[b] degrees of freedom between groups.
