# Supplementary material for: Biocontrol Potential of Raw Olive Mill Waste Against Verticillium dahliae in Vegetable Crops
Source: Plants (Basel). 2025 Mar 10;14(6):867. doi: 10.3390/plants14060867 (PMC11944966; doi:10.3390/plants14060867)
Supplement: Supplementary file 1 [file plants-14-00867-s001.zip › Supplementary Tables/Supplementary Table S7.pdf]

Table S7. Primers, sequences and thermocycling conditions used to amplify the 16S rRNA and ITS2 genes for bacteria and fungi, respectively.

	Primer	PCR program	Primer Sequence	Reference
Bacteria	515f	98°C for 10s, 50°C for 30s, 72°C for 30s (25 + 7 cycles) **, 72ºC for 10 min	NNNNNNNNNN**GT**GTGYCAGCMGCCGCGGTAA*	[37]
	806r		GGACTACNVGGGTWTCTAAT	
Fungi	fITS7	98°C for 10s, 55°C for 30s, 72°C for 30s (25 + 7 cycles) **, 72ºC for 10 min	GTGARTCATCGAATCTTTG	[38]
	ITS4		NNNNNNNNNN**GA**TCCTCCGCTTATTGATATGC*	

* Multiplex primer sample – index primer (consecutive Ns) and the linker in bolt before the forward primer

** The first number in parentheses indicates the number of cycles performed in the first PCR where the primers without multiplexing were used, while the second number indicates the additional cycles performed in the multiplexing PCR.
